# Simultaneous CRISPR screening and spatial transcriptomics reveal intracellular, intercellular, and functional transcriptional circuits

**DOI:** 10.1016/j.cell.2025.02.012

**Published:** 2025-03-12

**Authors:** Loϊc Binan, Aiping Jiang, Serwah A. Danquah, Vera Valakh, Brooke Simonton, Jon Bezney, Robert T. Manguso, Kathleen B. Yates, Ralda Nehme, Brian Cleary, Samouil L. Farhi

**Affiliations:** 1 Spatial Technology Platform, Broad Institute of MIT and Harvard, Cambridge, MA 02142, USA; 2 Klarman Cell Observatory, Broad Institute of MIT and Harvard, Cambridge, MA 02142, USA; 3 Cancer Program, Broad Institute of Harvard and MIT, Cambridge, MA 02142, USA; 4 Krantz Family Center for Cancer Research, Massachusetts General Hospital, Boston, MA 02144, USA; 5 Department of Medicine, Harvard Medical School, Boston, MA 02115, USA; 6 Stanley Center for Psychiatric Research, Broad Institute of MIT and Harvard, Cambridge, MA 02142, USA; 7 Faculty of Computing and Data Sciences, Boston University, Boston, MA 02215, USA; 8 Department of Biology, Boston University, Boston, MA 02215, USA; 9 Department of Biomedical Engineering, Boston University, Boston, MA 02215, USA; 10 Program in Bioinformatics, Boston University, Boston, MA 02215, USA; 11 Biological Design Center, Boston University, Boston, MA 02215, USA

## Abstract

Pooled optical screens have enabled the study of cellular interactions, morphology, or dynamics at massive scale, but they have not yet leveraged the power of highly plexed single-cell resolved transcriptomic readouts to inform molecular pathways. Here, we present a combination of imaging spatial transcriptomics with parallel optical detection of *in situ* amplified guide RNAs (Perturb-FISH). Perturb-FISH recovers intracellular effects that are consistent with single-cell RNA-sequencing-based readouts of perturbation effects (Perturb-seq) in a screen of lipopolysaccharide response in cultured monocytes, and it uncovers intercellular and density-dependent regulation of the innate immune response. Similarly, in three-dimensional xenograft models, Perturb-FISH identifies tumor-immune interactions altered by genetic knockout. When paired with a functional readout in a separate screen of autism spectrum disorder risk genes in human-induced pluripotent stem cell (hIPSC) astrocytes, Perturb-FISH shows common calcium activity phenotypes and their associated genetic interactions and dysregulated molecular pathways. Perturb-FISH is thus a general method for studying the genetic and molecular associations of spatial and functional biology at single-cell resolution.

## In brief

Perturb-FISH, a method that combines high-throughput imaging spatial transcriptomics with *in situ* guide RNA detection, allows for the analysis of molecular and functional states, with spatial resolution at the single-cell level.

## Graphical abstract

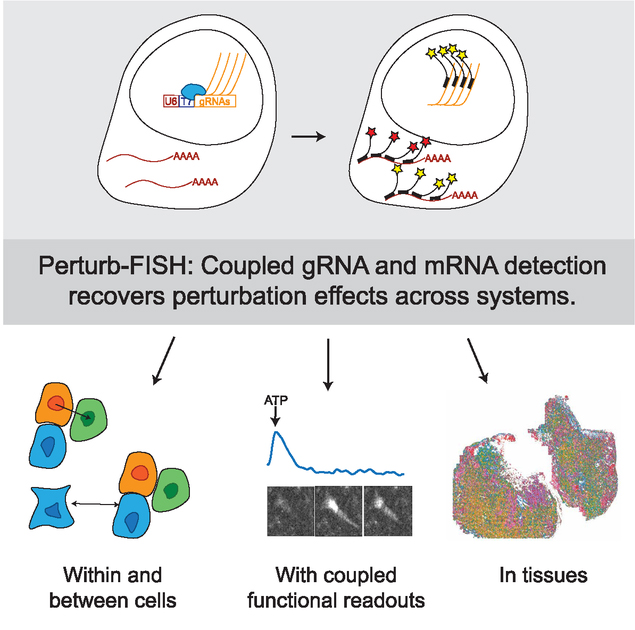

## INTRODUCTION

Pooled loss-of-function screening has proven to be a powerful application of CRISPR-Cas9 technology, identifying the genetic underpinning of phenotypes such as enhanced growth,^1^ drug resistances,^2^ and virus infection rates.^3^ The scalable experimental workflow of pooled guide RNA (gRNA) cloning, lentiviral packaging, and subsequent sequencing-based gRNA readout allows for high-throughput experiments where large numbers of genes are perturbed. More recently, pooled screening has been combined with single-cell RNA sequencing (scRNA-seq) to combine suppression of target genes with transcriptome-wide expression measurements in the same cell.^4–6^ This has allowed for mapping gene circuits within cells and for determining causal connections between the expression of a gene and that of downstream pathways. However, these approaches share the limitations of dissociated scRNA-seq measurements: namely, that the cell’s local context is lost. Thus, although it is known that gene expression in a given cell is affected by its direct neighborhood,^7,8^ these effects cannot be determined from droplet-based CRISPR screens. To decipher intercellular genetic interactions, one needs to be able to record gene expression and gRNA perturbations in their spatial context.

Optical pooled screening provides an alternative to sequencing-based pooled screens by combining *in situ* gRNA sequencing with imaging-based profiling. These methods are extremely high throughput but have not been linked to gene expression measurements.^9–11^ Incorporating spatial transcriptomic measurements of *in situ* gene expression profiles would allow not only probing gene regulatory circuits at both intracellular and intercellular levels but also pairing such data with functional measurements of the system, such as time-lapse recording of the response of cells to a given stimulus. Dhainaut et al. have demonstrated a combination of gRNA perturbation and sequencing-based spatial transcriptomics in Perturb-map, by using protein barcodes of gRNAs paired with 10x Visium to spatially readout perturbation effects.^12^ While this approach successfully pairs transcriptomics, CRISPR perturbation, and spatial information, the use of protein barcodes sacrifices the advantages of pooled cloning workflows, and the spatial resolution of Visium limits the use of the approach on cells that do not grow clonally. There is thus still a need for a tool that combines the benefits of single-cell spatial measurements with *in situ* gRNA readout at scale.

**Figure 1.**
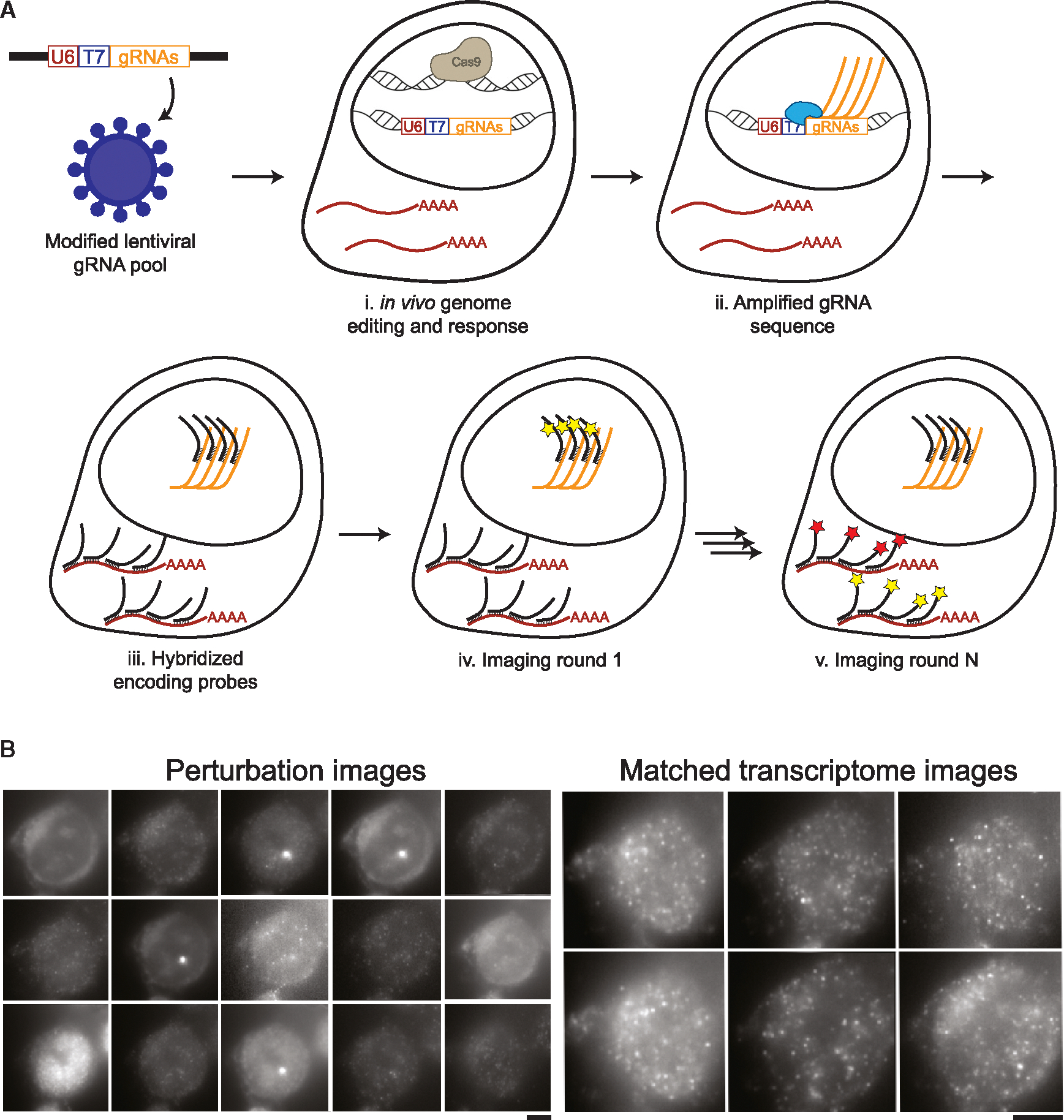
Perturb-FISH allows recording both gRNAs and transcriptome in cells in their spatial context (A) Perturb-FISH workflow. (Ai) Guides are packaged into lentiviral particles using a modified version of lentiguide-puro that contains a T7 promoter between the end of the U6 promoter and the beginning of the guide. (Aii) The lentivirus is used to insert the guide sequence in the genome of the cell, resulting in genome editing. (Aiii) T7 polymerase locally generates many copies of the gRNA in fixed cells. (Aiv) DNA-encoding probes anneal on both the target mRNA and the amplified gRNA. (Av) Fluorescent readouts anneal on the encoding probes and are imaged. The fluorophores are cleaved, and this step is repeated. (Avi) After sequentially imaging the gRNAs, rounds of hybridization/imaging/cleavage continue to image the transcriptome in the same cells. (B) Representative images of Perturb-FISH: amplified gRNA generates a bright spot in the nuclei of cells, and the identity of gRNAs is encoded in the sequence of images in which they fluoresce (left). The transcriptome is read out the same way with MERFISH (right). T7 transcription yields higher signal amplification than the tiling of an mRNA with 30 probes, as visible by the larger size of the spots they generate. Scale bar, 10 μm.

To meet this need, we turned to imaging spatial transcriptomics (iST) for its resolution, efficiency, scalability, and compatibility with home-brewed reagents at low cost. However, any technology that probes both transcriptome and gRNAs using microscopy needs to address a major challenge: identifying single molecules over background autofluorescence, which requires signal amplification. Multiplexed error robust fluorescence *in situ* hybridization (MERFISH) achieves this for mRNA measurements by tiling each mRNA with a minimum of 25 “encoding” probes, each of them annealing on a 30-base-pair (bp) region of the target molecule.^13,14^ These barcoded encoding probes are then read out over multiple rounds of staining, imaging, and destaining to recover mRNA identity. The barcodes are structured with a Hamming weight of 4, meaning that individual molecules light up in 4 of the images acquired, and Hamming distance of 4, meaning that errors in binding can be identified and corrected computationally. Although this strategy yields single-molecule detection, gRNAs cannot be tiled in the same way as they are only 20 bp long. MERFISH was previously used in conjunction with CRISPR perturbations using a long barcode and a reporting gene,^15^ but this approach is limited by the challenges of pooled cloning of long barcodes, lentivirus packaging efficiency of long insert sequences, and lentiviral shuffling of barcodes distant from the gRNA^10,16^ In addition, the branched amplification readout strategy used in this work was noisy and required the expression of a reporter RNA, further complicating the experimental implementation. We reasoned that to leverage barcoded MERFISH readouts while allowing pooled workflows on gRNAs, gRNAs can be used as self-barcodes if adequately amplified *in situ*. Rather than tiling, we used an alternative approach for amplification by generating many local copies of the target molecule, by *in situ* transcription via T7 polymerase^17^ (Figure 1).

**Figure 2.**
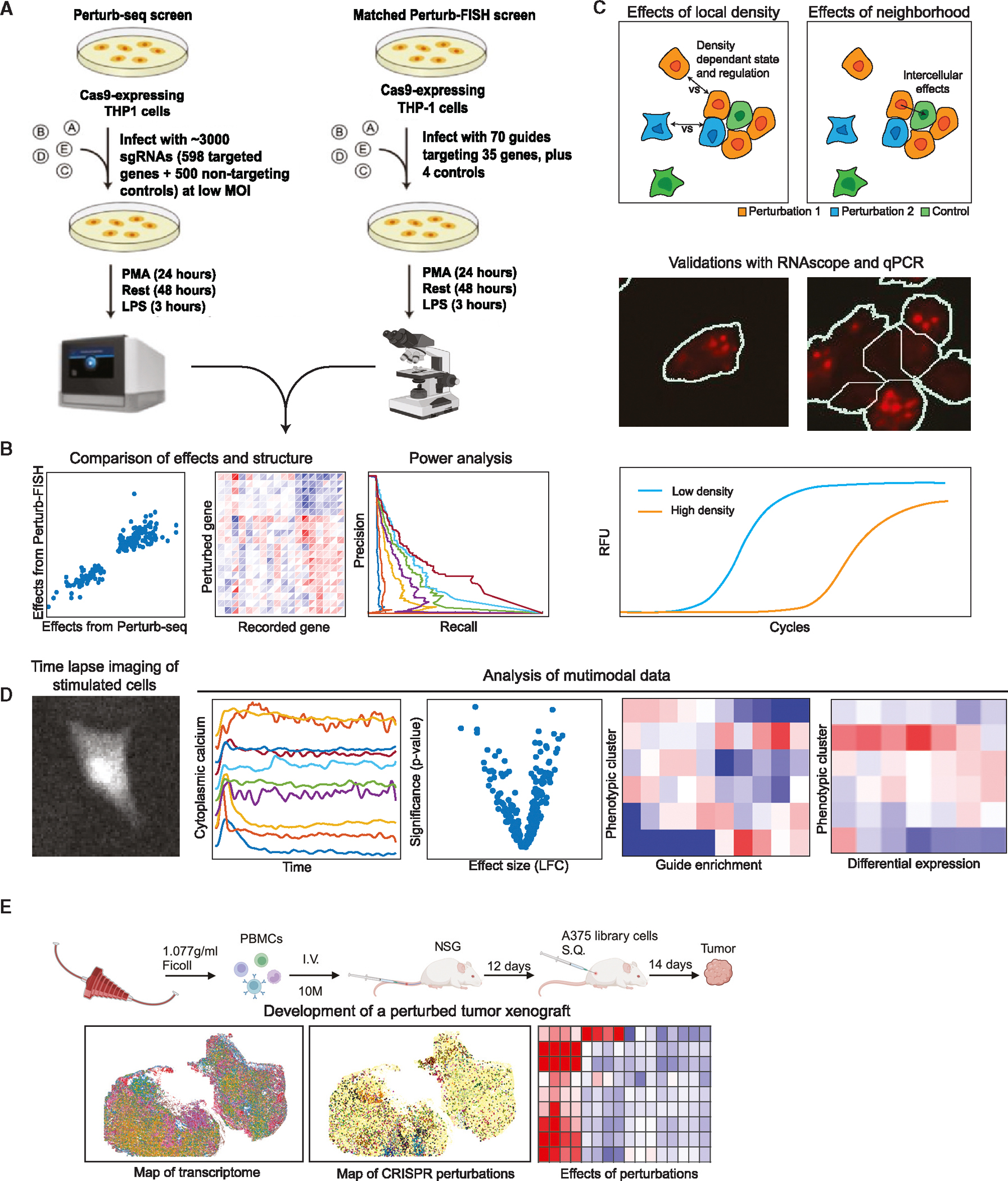
Overview of experimental design and analysis (A) Overview of matched Perturb-seq and Perturb-FISH screens for genetic regulators of LPS response in THP1-cells. (B) We compare perturbation effect sizes from the two screens to evaluate Perturb-FISH in terms of consistency, recapitulation of global regulatory structure, and power (Figure 3). (C) We first characterize the effects of local cell density on perturbations effects, then we recover effects from perturbed cells onto their unperturbed control neighbors. We validate these results using both qPCR and RNAscope (Figure 4). (D) We demonstrate the use of Perturb-FISH in conjunction with live imaging characterization of calcium phenotypes, in a screen of ASD risk genes (Figure 5). (E) We verify that Perturb-FISH can be applied in 3D tissues, in a screen of NF-κB genes in tumors.

Here, we introduce perturbation-based fluorescence *in situ* hybridization (Perturb-FISH), which combines MERFISH with local amplification of the gRNA region, to decode both perturbations and transcriptome in their spatial context. We demonstrate Perturb-FISH in a system for which we had previously generated matched data using perturbation-based single-cell RNA sequencing (Perturb-seq) (Figure 2A): the genetic network regulating the response of THP1-derived macrophages to lipopolysaccharide (LPS) stimulation.^18^ We validated Perturb-FISH by comparing the CRISPR knockout (KO) effects of gRNA perturbation as recovered by Perturb-FISH with those recovered by Perturb-seq (Figure 2B). We then demonstrated the power of Perturb-FISH to interrogate intercellular genetic circuits by examining how cell density (Figure 2C) impacts the effects of gRNA perturbations and how cells are affected by the presence of certain perturbations in their direct neighbors. Finally, we extended Perturb-FISH by coupling a functional readout in a CRISPR inhibition (CRISPRi) screen of autism spectrum disorder (ASD) risk genes. We record knockdown (KD) effects on both calcium activity (assayed via live imaging) and gene expression in human-induced pluripotent stem cell (hIPSC)-derived astrocytes (Figure 2D). Finally, we demonstrate the applicability of Perturb-FISH in 3D tissues by recording the effects of KO perturbations in a tumor xenograft, and we interrogate the genetic interactions between tumor cells and infiltrating lymphocytes.

## RESULTS

### Perturb-FISH allows recording both gRNA and mRNA *in situ*

We modified a lentiviral vector commonly used to transduce cells with gRNAs (lentiguide-puro)^19^ to include a T7 phage promoter between the end of the U6 promoter (responsible for the expression of gRNAs in the live cell) and the beginning of the gRNA sequence itself (Figure 1Ai). As shown by Romanienko et al.,^20^ this fused U6T7 promoter retains its capacity to express functional gRNAs *in vivo* through activation of the U6 promoter, while also allowing *in situ* amplification in fixed cells using the T7 promoter. Because transcription from the U6 promoter starts 23 bases after the end of the essential sequence, the T7 sequence is not transcribed with the guide. Local amplification of DNA from a T7 promoter is used in the Zombie is optical measurement of barcodes by *in situ* expression (ZOMBIE) protocol^17^ to allow for decoding of single-cell barcodes *in situ*.

In Perturb-FISH, this *in situ* transcription protocol (Figure 1Aiii) is followed by a proteinase-based clearing step and a standard MERFISH protocol involving staining with a pre-designed encoding probe library containing probes against both guides and mRNAs of interest (Figure 1Aiv). During imaging, 20 bp fluorescent imaging, or “recording,” probes are sequentially flowed onto the sample and imaged^13,14^ (Figures 1Av and 1Avi). Between each round, the fluorophore is cleaved off from the readout probes by disulfide reduction. To make all chemistries compatible, incubation temperatures for both gRNA- and mRNA-encoding probes must be similar. We found that the 20-bp gRNA length was too short: the melting temperature of 20-bp gRNA-encoding probes was lower than the temperature of the formamide washes that are part of the MERFISH protocol. We extended the target region into the first ten bases of the gRNA scaffold, which allowed us to record multiplexed data for both gRNAs and mRNAs. High multiplexing is achieved using the same approach as MERFISH to encode the identity of each perturbation: each RNA molecule is assigned a sequence of 4 (in this case out of 15) images in which it is “on” (Figure 1B). Computational reconstruction registers images, assigns barcodes to locations, performs error correction built into the library design, and finds gRNA and mRNA identities vs. spatial locations (STAR Methods). The images are then segmented to yield count tables of mRNA expression and gRNA identity.

### Perturb-FISH recovers genetic perturbation effects with high correlation both intrinsically and with Perturb-seq

We first applied Perturb-FISH to a system for which we have matched data from Perturb-seq: the response of THP1-derived macrophages to (LPS) stimulation.^18^ Cells were perturbed using a library of 74 gRNAs against 35 target genes, differentiated into macrophages, and then stimulated with LPS (Figure 2A). After images were decoded and the data condensed in count tables, the effect of each perturbation was computed using FR-Perturb, a method we recently developed to infer perturbation effects.^18^ We generated replicate datasets from two coverslips to assess reproducibility.

**Figure 3.**
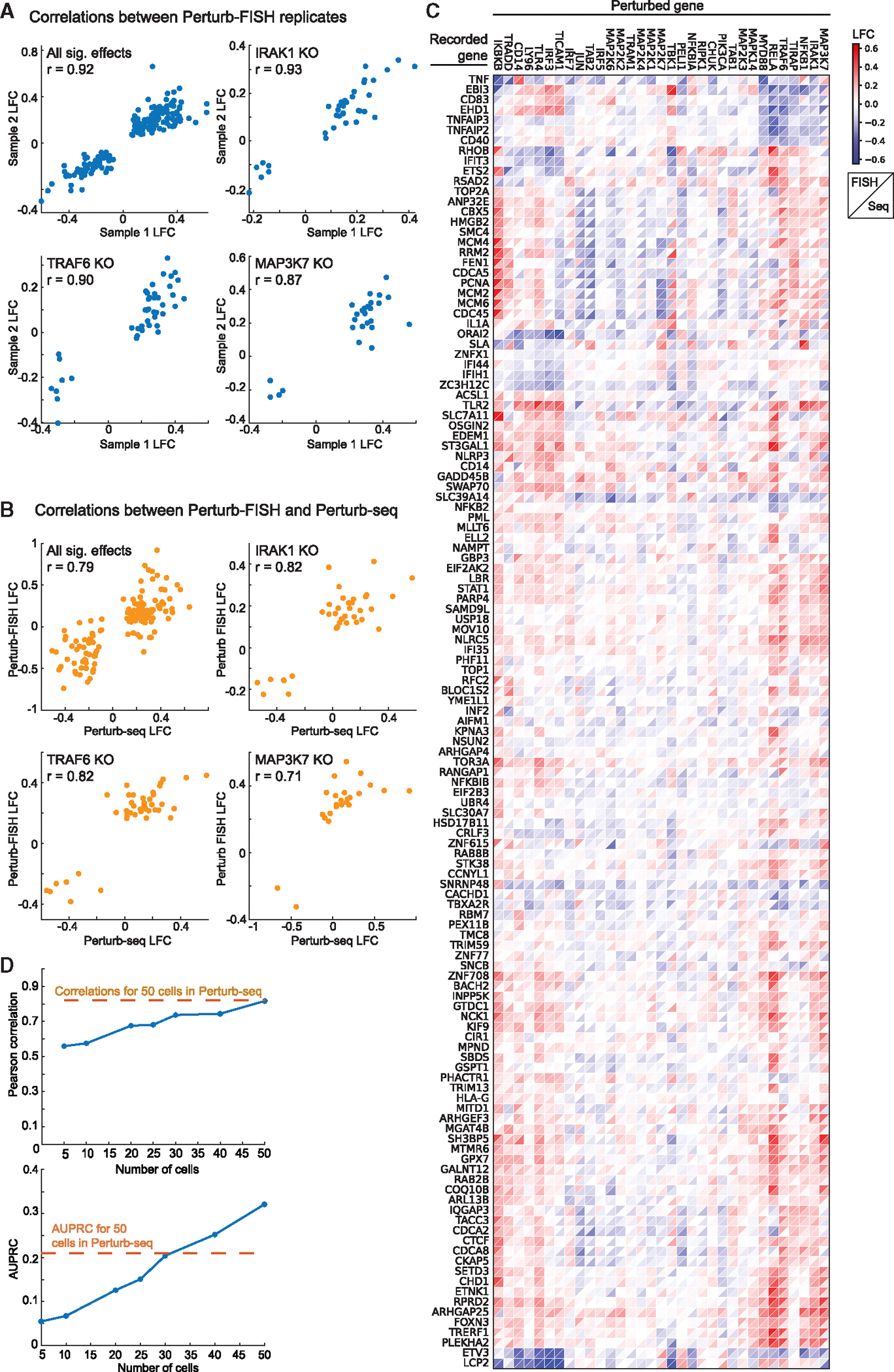
Perturb-FISH robustness and power analysis (A) Scatterplots of significant effect sizes (*q* < 0.1) determined in Perturb-FISH sample 1 (x axis) and the same effect sizes as determined in sample 2 (y axis). Effects represent log-fold changes (LFCs; natural log base) in expression relative to control cells. Individual plots depict significant effects across all perturbations or for individual perturbations *MAP3K7*, IRAK1, and TRAF6. (B) Scatterplots of significant effect sizes (*q* < 0.1) determined in Perturb-seq (x axis) and combined analysis of both Perturb-FISH replicates (y axis). Effects represent LFCs(natural log base) in expression relative to control cells. Individual plots depict significant effects across all perturbations or for individual perturbations *MAP3K7*, *IRAK1*, and *TRAF6*. (C) Heatmap of LFC effect sizes (color bar) found in Perturb-FISH (upper left triangle of each square) and the same effects in Perturb-seq results (lower right triangle). Rows and columns are clustered based on unweighted pair group method with arithmetic mean (UPGMA) clustering of Perturb-FISH data. (D) Held-out validation accuracy (y axis; Pearson correlation, left; AUPRC, right) of effects learned in downsampled Perturb-FISH data using increasing numbers of cells (x axis). Orange line shows approximate correlation (left) or AUPRC (right) obtained with 50 cells in Perturb-seq, estimated using previously published data.^18^ See also Figure S1.

The inferred effects were self-consistent within Perturb-FISH experiments and highly concordant with Perturb-seq results. Effects of perturbations for key genes (*MAP3K7*, *IRAK1*, *TRAF6*, *RELA*) of the nuclear factor κB (NF-κB) pathway were highly correlated between the two Perturb-FISH samples (Pearson correlation coefficient of significant effects from Perturb-FISH replicates of 87%, 93%, 90%, 85% respectively), with all statistically significant effects across perturbations being 92% correlated (Figures 3A and S1A). Significant effects for those four genes are also highly correlated between Perturb-FISH replicates and the matched Perturb-seq data, with respective Pearson correlations of 87%, 78%, 82%, and 75% between sample 1 and Perturb-seq and 68, 79, 87, and 80 between sample 2 and Perturb-seq (Figure 3B). We further validated our results by separately inferring the effects of each guide targeting the same gene (two guides are present for each gene in the library) and found that effects remain highly correlated (Figure S1B). Since data from both Perturb-FISH replicates were highly correlated, we pooled them together to increase statistical power for the rest of the study. After pooling, the correlation between pooled Perturb-FISH data and Perturb-seq data remains high at 79% for effects significant in both modalities. As expected from the overall agreement in individual perturbation effects, the global structure of perturbation effects is largely consistent between modalities: both extract the same modules of target genes that affect cells in a similar way when perturbed, as well as comparable programs of genes that react similarly to a given perturbation (Figure 3C). These clusters match previous knowledge of the system, with tumor necrosis factor (*TNF*), *TNFAIP2*, and interleukin-1a (*IL1A*) being among the genes most downregulated by perturbing targets such as *MAP3K7*, *IRAK1*, *TRAF6*, *RELA*, and *MYD88*.^18,21,22^

While testing the consistency of Perturb-FISH data, we noticed a few outlier effects that correlate between Perturb-FISH datasets but do not correlate with Perturb-seq (Figure S1C). These come from perturbations *IRF3*, *TLR4*, and *CD14* that showed no effect in Perturb-seq but gave significant effects in Perturb-FISH. Notably, significant effects from these perturbations are highly concordant between Perturb-FISH datasets, and they agree with the current understanding of the pathway as the KO of the main LPS receptor *TLR4* is expected to downregulate genes from the immune response like *TNF* or *IL1A*.^23,24^ Similarly, *CD14* KO^25^ and *IRF3* KO^26^ were both shown to upregulate *TNF* expression in response to LPS.

Next, we ran a power analysis to identify the minimal number of cells per target to get reliable effects by Perturb-FISH. To do so, we reserved half of the data as a validation set, and we used the other half to test the consistency of the inferred effects as we increased the number of cells used for each perturbation, from 5 to 50 cells. Plotting only the effects that are statistically significant according to the validation set, we observed that correlation of effects sizes between subsampled test set and validation set steadily increases with the number of cells used in the smaller subset, from 68% with 20 cells to 74% with 30 and 40 cells, and finally reaching 81.7% with 50 cells, which is comparable to what we achieved with our matched Perturb-seq study (Figure 3D). For all sizes, considering only the effects that are statistically significant according to both the test set and the validation set gives correlations above 98%. Finally, we built precision/recall curves and calculated area under the precision/recall curve (AUPRC) (Figures 3D and S1D), which showed improved precision and recall (starting around 30 cells), compared with matched Perturb-seq data.^18^ Therefore, Perturb-FISH allows making hypotheses on perturbations effects with power comparable to Perturb-seq using 20–50 cells per perturbation target.

### Perturb-FISH recovers cell-extrinsic effects of gene perturbation

**Figure 4.**
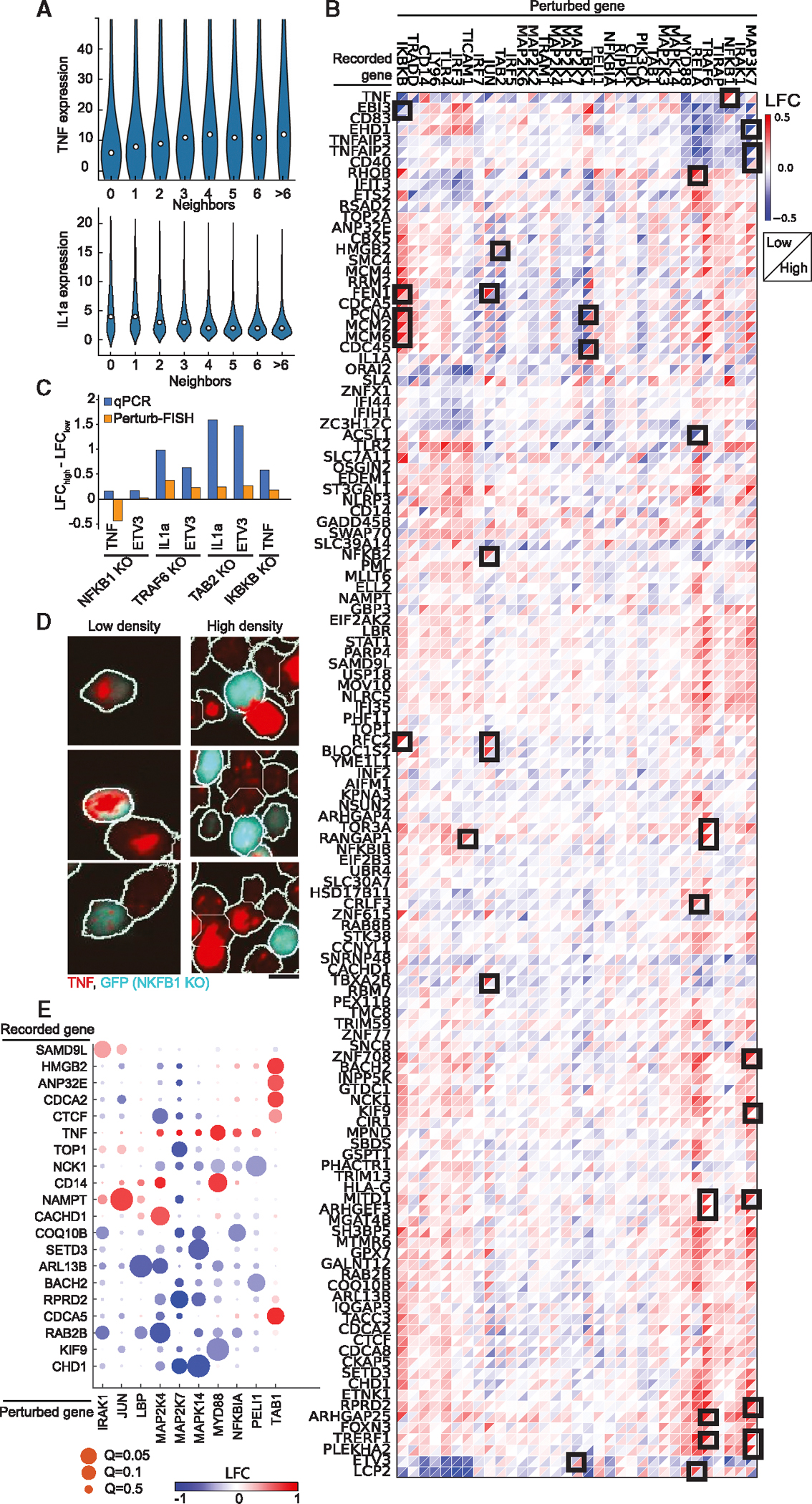
Perturb-FISH analysis of density-dependent and intercellular effects (A) Violin plots show the expression level (y axis) of *TNF* (top) and *IL1A* (bottom) in cells with different numbers of neighbors (x axis). (B) Heatmap of LFC effect sizes (color bar) found by Perturb-FISH in cells with two or fewer neighbors (upper left triangle of each square) or three or more neighbors (lower right triangle). Effects represent log-fold changes (LFCs; natural log base) in expression relative to control cells at matching density. Black squares highlight effects that are significant (*q* < 0.1) in at least one density and different by at least 0.4 between densities. (C) Comparison of the density dependency of the effects of perturbing *NFKB1*, *TRAF6*, *TAB2*, or *IKBKB* on *TNF*, *ETV3*, or *IL1A* expression. Bars show the difference between effects at high density and effects at low density, when effects are measured with Perturb-FISH (in orange) or with a qPCR (blue). Effects are expressed as LFCs (natural log base). (D) RNAscope images showing *TNF* transcripts (red) in *NFKB1* KO cells (blue) and unperturbed cells (black) at both low density (left) and high density (right). (E) Heatmap of LFC effect sizes (color bar) of gene knockouts (x axis) on the expression of genes (y axis) in neighboring unperturbed cells. Effects are expressed as LFCs (natural log base). Circle size shows significance, with *q* values from 0.05 to 0.5 s. See also Figure S2.

The previous data highlight that Perturb-FISH recovers the intracellular effects of genetic perturbations at least as well as Perturb-seq. We next investigated the additional information that is afforded by an *in situ* approach, in particular the effects of local cell density on gene expression. When grown on a glass surface, THP1s will spontaneously organize in areas of variable cell density (Figure 2C). We observed that the expression of certain genes has a high dependency on cell density. In particular, expression of hallmark LPS response genes *TNF* and *IL1A* varied considerably with the number of immediate neighbors to a cell (Figure 4A), with *TNF* raw counts increasing up to 1.34-fold with the number of neighbors and *IL1A* raw counts decreasing up to 2.6-fold. The expression levels of *TNF* and *IL1B* have been previously reported to depend on cell density, although the directionality of the effect is inconsistent between studies.^27–30^

In light of these results, we analyzed the Perturb-FISH data to identify density-dependent effects of genetic perturbation. To do so, we split the data into one set of cells at low local density (with two or fewer contacting neighbors) and another set of cells at high density (three or more contacting neighbors). While dividing the datasets brought the analysis close to the limit of the statistical power of this dataset, we discovered that while most perturbations showed concordant effects at the two densities, many perturbations have substantially different effects depending on cell density (Figure 4B). Of note, *NFKB1* KO results in an upregulation of *TNF* only in cells at low density (log-fold change at low density [LFC_low_]: 0.47, *Q*: 0.014, LFC_high_: 0.03). Meanwhile, *TRAF6* KO results in a much stronger *IL1A* downregulation at low density (LFC: −0.44, *Q*: 0.14) than it does at high density (LFC; −0.06, *Q*: 0.7) (Figure 4B). *ETV3* is another example of a gene that is further downregulated at low density compared with high density by both *TRAF6* KO (LFC_low_: −0.38, LFC_high_: −0.17) and *NFKB1* KO (LFC_low_: −0.53, LFC_high_: −0.27). Note that LFCs in this text use a natural log basis.

We used two approaches to validate these results. We first conducted a qPCR experiment in which we grew THP1 cells as a mix of 14% perturbed cells (for either *NFKB1* or *TRAF6* and labeled with GFP in each case) with non-perturbed controls and seeded in different wells with two densities (low density: 25,000 cells/cm^2^, high density: 216,000 cells/cm^2^). After LPS stimulus, we sorted perturbed and control cells from each density by fluorescence-activated cell sorting (FACS) and extracted their RNA to quantify the expression levels of genes of *TNF*, *IL1A*, and *ETV3*. Most differences of effect sizes are concordant between qPCR and Perturb-FISH (Figure 4C), with the notable exception of the effect of *NFKB1* on *TNF*. The dependency of bulk *TNF* expression with average cell density has been reported before, albeit with inconsistent directions.^27,28^ We therefore used RNAscope^31^ to better characterize this effect at the single-cell level since it is one of the perturbations most affected by density according to Perturb-FISH (LFC_low_: 0.45, *Q*: 0.015, LFC_high_: 0.007). We reasoned that an assay that does not require averaging expression across cells that are exposed to varied densities in a dish would have better sensitivity. We find that RNAscope does confirm the Perturb-FISH findings: *NFKB1*-KO cells overexpress *TNF* at low density but repress it at high density (Figures 4D, S2A, and S2B). We also used RNAscope to validate the finding that *IL1A* is expressed at lower levels in macrophages at high density (Figure S2C). RNAscope data show that *TRAF6* KO causes a complete silencing of *IL1A* regardless of local cell density (Figure S2D). Therefore, the density-related difference in effects evaluated with Perturb-FISH originates from the density-dependent difference in expression of *IL1A* in control cells, as this changes the value used as a reference in the comparison. Overall, we find that Perturb-FISH uncovers density-related effects of genetic perturbations, which are validated by other experimental approaches. These effects highlight the existence of mechanisms that regulate immunity at a cell population level and could be relevant to diseases related to overactivation or underactivation of the immune response and inflammation.

Next, we sought to identify intercellular effects of genetic perturbation on gene expression in neighboring cells. We analyzed cells that received a control guide and looked for effects coming from perturbed neighbors. We find that nine perturbations have significant effect on gene expression in their control neighbor cells, including *MYD88* (Figure 4E). Cells with a *MYD88* KO provoke an increase of *TNF* (LFC: 0.83, *Q*: 0.06) and *CD14* (LFC: 0.77, *Q*: 0.018) in their neighbor. *MAP2K7* perturbation also has several effects in neighboring cells, downregulating *TOP1* (LFC: −0.75, *Q*: 0.06), *RPRD2* (LFC: −1.01, *q*: 0.028), and *CHD1* (LFC: −1.08, *Q*: 0.06). While this analysis does not reach statistically significant effects from *IRF3* perturbation on *IL1A*, it suggests that *IL1A* is overexpressed in cells that neighbor an *IRF3* KO cell (LFC: 1.65, *Q*: 0.7), which is validated by our RNAscope data (Figures S2C and S2D).

These results reveal co-regulatory gene networks between macrophages responding to a bacterial infection and highlight the capacity of Perturb-FISH to interrogate genetic interactions between cells, which we believe will be a key tool in understanding how multicellular circuits underlie tissue function.

### Perturb-FISH for functional screens: Interrogating ASD risk gene networks in disrupted calcium activity

To demonstrate the ability of Perturb-FISH to match perturbation data with functional imaging, we then applied it to study ASD risk genes and their role in regulating gene expression and calcium activity in iPS-derived astrocytes. Whole-exome sequencing studies have recently identified a large number of highly penetrant but rare risk genes associated with ASD, but little is known about their molecular or phenotypic role, especially in the brain. A tempting hypothesis is that these risk genes converge on a smaller set of molecular and cellular pathways.^32^ While expression data have shown that ASD risk genes are enriched in neuronal cell types, several publications have highlighted transcriptional differences within astrocytes between ASD patients and control groups.^33–35^ There have also been reports of a functional role of calcium activity in the development of ASD symptoms,^36–38^ specifically that ASD patient iPS-derived astrocytes show aberrant calcium activity and that injecting these cells into healthy mice is enough to generate ASD-like behaviors^39^ or that disrupting calcium activity in astrocytes generates ASD-like behaviors in mice.^38^ Finally, a study has reported the first enrichment for regulons downstream of several ASD risk genes in astrocyte subtypes.^40^ We thus decided to explore the power of Perturb-FISH to study an unexplored biological problem by determining if a set of ASD risk genes with largely unknown roles converge on aberrant calcium signaling in iPS-derived astrocytes.

We designed a Perturb-FISH screen to determine if knocking down 127 highly penetrant ASD risk genes^41^ (STAR Methods) could directly lead to dysfunction of calcium activity in astrocytes differentiated from the UCSFi001-A line (human pluripotent stem cells from healthy male donor), stably expressing dCas9-KRAB. This line was previously used for CRISPRi-based expression and morphology screens.^42^ Calcium transients have been well characterized to follow ATP stimulation in astrocytes,^43–45^ through *ITPR*-mediated release of calcium ions from the endoplasmic reticulum (ER), and therefore we also included gRNAs against *ITPR3* as positive controls.^46^ The genes selected for expression measurements included the perturbed targets and a published list of 358 differentially expressed genes between astrocytes from ASD and control patients.^34^

**Figure 5.**
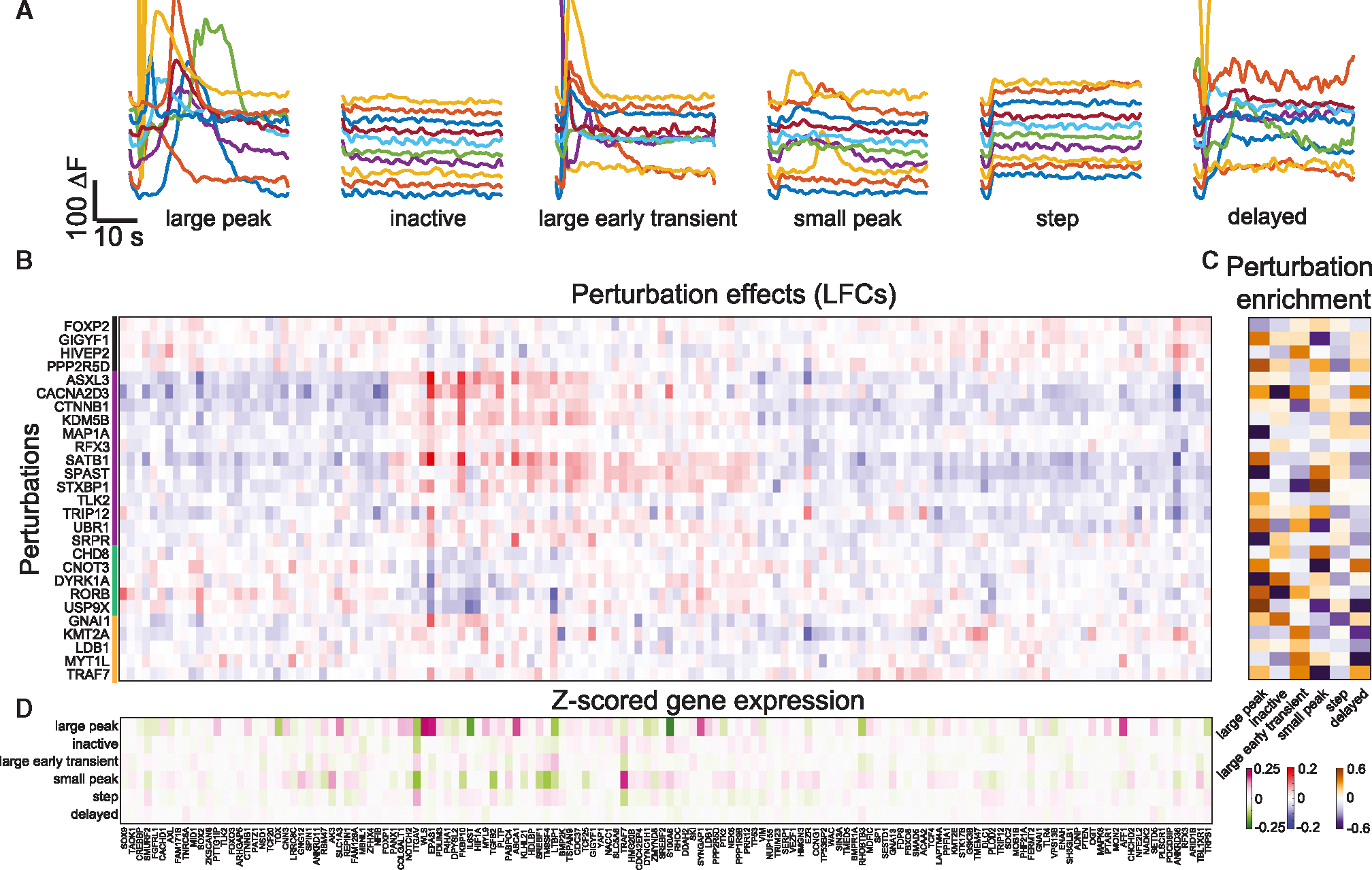
Perturb-FISH analysis of the role of ASD risk genes in generating different calcium activity phenotypes in astrocytes (A) Traces showing the variation in cytoplasmic calcium (y axis) concentration in example cells (line) across time (x axis), following ATP stimulation. Variation in calcium concentration shown as difference in fluorescence. Cells are six groups using k-means clustering on features extracted from the traces: large peaks; inactive cells; cells with large early transients; cells with a small peak; cells with a step; and finally, cells with a delayed response. (B) Heatmap of LFC effect sizes (color bar) found by Perturb-FISH in iPSC-derived astrocytes. Perturbations on the y axis; genes (x axis) are aligned with and labeled on (D). (C) Heatmap of LFC perturbation enrichment (y axis, aligned with B, color bar) per calcium activity signature (x axis). (D) Heatmap showing genes with differential expression (x axis, aligned with B, color bar) and their *Z* scored expression (scale bar) in six clusters of calcium activity. See also Figures S3 and S4.

To characterize functional profiles, we stimulated astrocytes with 250 μM ATP and recorded calcium activity in 76,400 cells from 3 pooled replicates (Figure 2D). We parametrized the calcium traces for every cell (STAR Methods) to identify cells that responded to the stimulation by starting calcium oscillations. As shown previously by our group and others,^47,48^ this parameterization allows for clustering of astrocytes in several groups with or without transients of several groups, which we annotated as follows: “large peak”; “inactive”; “large early transient”; “small peak”; “step”; and finally, “delayed” based on their calcium temporal dynamics (Figures 5A and S3). Notably, the cluster of cells’ large peak best resembles the activity pattern found in ASD astrocyte lines in a previously reported ASD case-control cohort.^39^

The simultaneous mRNA expression readout of Perturb-FISH allows the identification of expression profiles that are associated with each functional cluster (Figure 5D). Within each cluster, 2–5 genes were upregulated, and 17–30 genes were downregulated. The large peak cluster showed the largest magnitude expression changes, including a downregulation of *S100A6*, *IL6ST*, and *LTBP1*, implicated in calcium binding^49–51^; and expression changes in multiple genes associated with altered synaptic function such as *SYNGAP1*, *SLC1A3*, *EPAS1*,^52^ *WLS*,^53^ and *ABCA1*.^54^

We then examined genetic factors that may drive these differences by quantifying gRNA enrichment per cluster (Figure 5C) and calculating a *p* value of being enriched or depleted. We again randomly split the dataset and verify consistency of the enrichment/depletion between each half (r = 0.75, Figure S4). Overall, astrocytes with control perturbations exhibit all calcium response patterns, matching our previous knowledge of the system,^55–57^ but perturbations to certain ASD risk genes bias cells to (or away from) specific functional profiles. Consistent with the idea that groups of ASD risk genes might act through similar phenotypic mechanisms, we found groups of perturbations significantly enriched in each functional cluster. For example, the large peak cluster that showed altered synaptic gene expression levels also showed significant depletions for KDs of *MAP1A*, *SPAST*,^58^ and *DYRK1A*— all previously connected with synaptic function—and an enrichment for KDs of *USP9X* (LFC: 0.87, *p*: 0.03), which is involved in neuronal development but has not yet been linked to astrocytic calcium signaling.

Finally, Perturb-FISH allows connecting the downstream expression of genes affected by each perturbation with the observed expression differences between functional phenotypes. This allows nominating molecular mechanisms that each perturbed gene regulates and how they induce different functional phenotypes. After quality control (QC) filtering (STAR Methods), we used FR-Perturb to infer perturbation effects. As above, we validated the consistency of effects by comparing correlations of effect between randomly split halves of the data: we find that significant effects remain highly correlated (Pearson r = 0.83 when considering all effects significant in at least one half, 0.94 when considering effects significant in both halves; Figure S4A). We also compare effects between experimental replicates: effects are on average 87% correlated between replicates when considering effects significant (*q* < 0.1) in both replicates and 62% correlated when considering effects significant (*q* < 0.1) in at least one of the replicates (Figure S4B). For the rest of the analysis, data from all replicates are pooled (STAR Methods).

We find 566 effects with a significant q value below 0.1, with 27 perturbations having more than 3 significant effects and 142 genes expression levels significantly altered by at least 3 perturbations. The perturbed genes could be clustered into four modules of ASD risk genes with similar downstream targets and five co-regulated modules of expressed genes affected in similar ways by the same perturbations (Figure 5B). Interestingly, the second co-regulated module of expressed genes includes a large number of cholesterol-associated genes, including *ABCA1*, *SREBF1*, and *HDLBP*. Recent scRNA-seq studies in ASD and schizophrenia both reported altered cholesterol biosynthesis gene expression,^40,59^ and these results suggest that ASD risk genes can regulate this gene expression module even in the absence of neurons.

While we expect that most perturbation effects reflect regulation in committed astrocytes, we also asked whether the observed expression changes could be due to a change in cell lineage commitment. We note that perturbations are only introduced 24 days into the differentiation protocol, 6 days before imaging, ensuring that cells have had time to develop an astrocytic signature at the beginning of the experiment. Nevertheless, three perturbations provoke a significant downregulation of *VIM* or *SOX9*, which are signatures of an astrocytic lineage. *ASXL3* KD downregulates both (LFC: −0.08 and −0.03, respectively), *CTNNB1* downregulates *SOX9* expression (LFC: −0.05), and *KDM5B* downregulates *VIM* expression (−0.07). Interestingly, *RORB* KD upregulates *SOX9* (LFC: 0.11). All other perturbations do not affect expression levels of these genes, and therefore cells indeed present an astrocytic phenotype, further confirmed by examining cell morphology (Figure S4D). This also suggest that the mechanisms by which these three genes participate in the development of ASD could be related to cell-type differentiation during brain development, specifically by favoring neuronal over glial lineages.

Finally, we show how all Perturb-FISH can be used to explore a dataset across all three modalities. The step cluster is depleted for *TRIP12* KD (LFC: −0.29, *p*: 0.02) and shows upregulation of *ITGAV* and *ZMYND8* and downregulation of *EZR* (*Z* scores: 0.11, 0.05, and −0.04, respectively). Consistently, the gene expression analysis shows that *TRIP12* KD causes downregulation of *ITGAV* and *ZMYND8* and upregulation of EZR (LFC: −0.06, −0.08, 0.1; *Q*: 0.05, 0.05, and 0.19, respectively). Inactive cells are enriched for *CREBBP* KD (LFC: 1.37, *p:* 0.01) while overexpressing the RAS homolog *RHOC* (*Z* score: 0.04), which is also overexpressed in response to *CREBBP* KD (LFC: 0.13, *Q:* 0.3). *CREBBP* KD is known to enhance the RAS/RAF/MEK/ERK pathway^60^ and was previously shown to play a role in calcium activity in astrocytes that form tripartite synapses around postsynaptic dendritic spines by mediating calcium activity through cyclic AMP.^61^

In the functionally interesting large peak cluster, we observe upregulation of *TRAF7* (*Z* score: 0.18), and consistently, we find that *TRAF7* KD cells are indeed depleted from this cluster (LFC: −0.9, *p*: 0.018). Following our observations of *USP9X* KD enrichment above, we also found that *USP9X* KD results in altered expression of *FAM126A*, *NACC1*, *P4HA1*, *DPYSL2*, *FKBP10*, *IL6ST*, and *LTBP1* (LFC_FAM126A_: 0.04, Q_FAM126A_: 0.3, LFC_NACC1_: 0.04, Q_NACC1_: 0.21, LFC_P4HA1_: −0.05, Q_P4HA1_: 0.06, LFC_DPYSL2_: −0.05, Q_DPYSL2_: 0.18, LFC_FKBP10_: −0.05, Q_FKBP10_ = 0.07, LFC_IL6ST_: −0.1, Q_IL6ST_: 0.0099, LFC_LTBP1_: −0.1, Q_LTBP1_: 0.13) and correspondingly observed altered expression in the functional cluster (*Z* scores 0.05, 0.07, −0.05, −0.07, −0.04, −0.17, and −0.125, respectively). Beyond *IL6ST* and *LTBP1’s* calcium binding roles,^49–51^ *DPYSL2* plays a role in the trafficking of calcium channels to the membrane,^62^ and *FKBP10* encodes for a protein localized in the ER that binds calcium.^63,64^ Most of these genes are involved in the mTOR pathway: *NACC1*,^65^ *FKBP10*,^66^ *DPYSL2*,^67^ *P4HA1*,^68^ *USP9X*.^69^ mTOR has been recently hypothesized to play a key role in the development of ASD,^70–73^ and our data agrees with previous literature^74,75^ showing that the mTOR pathway plays a role in astrocyte calcium function.

In the step cluster, we observe upregulation of *ITGAV* and *ZMYND8* (*Z* score: 0.11 and 0.05) and downregulation of *EZR* (*Z* score: −0.04). These three genes are all downstream of *TRIP12* KD, which causes the downregulation of *ITGAV* (LFC: −0.06, *Q*: 0.05), *ZMYND8* (LFC: −0.08, *Q*: 0.05) and the upregulation of *EZR* (LFC: 0.1, *Q*: 0.19). Consistently, *TRIP12* KD cells are depleted from the cluster step (LFC: −0.29, *p*: 0.02). The depletion of cells with this perturbation from the cluster is coherent with its effects on these two genes being opposed to their signature expression in that cluster. *CREBBP* KD cells are enriched in the cluster of inactive cells (LFC: 1.37, *p:* 0.01). The *RAS* homolog *RHOC* is overexpressed in the cluster of inactive: cells (*Z* score: 0.04). It is overexpressed in response to *CREBBP* KD (LFC: 0.13, *Q:* 0.3). Consistent with these results, *CREBBP* KD is known to enhance the RAS/RAF/MEK/ERK pathway^60^ and was previously shown to play a role in calcium activity in astrocytes that form tripartite synapses around postsynaptic dendritic spines by mediating calcium activity through cyclic AMP.^61^

### Perturb-FISH recovers effects of genetic perturbations in tissues: Interrogating the NF-κB pathway in perturbed human melanoma cells grafted in immunocompromised mice

To explore the Perturb-FISH’s utility in more complex tissue environments, we implemented it in a mouse xenograft model. We chose to study genetic perturbations to the NF-κB pathways because (1) the NF-κB pathway and inflammation play significant roles in the development of tumors,^76^ and (2) we were able to reuse the library from our THP1 screen, which was shown to produce significant and reproducible effects. We used the same set of perturbations to 35 target genes that were included in our THP1 screen above. We perturbed these 35 genes in A375 cells, a human melanoma cell line, and injected these cells into the flank of immunocompromised (NOD scid gamma [NSG]) mice 1 week after engrafting human peripheral blood mononuclear cells (PBMCs) from a healthy donor (Figure 2E). Tumors were allowed to grow for 2 weeks, before extraction and snap freezing. As a readout, we measured expression of 500 genes from an immune-oncology panel that records the immune response to cancer.

**Figure 6.**
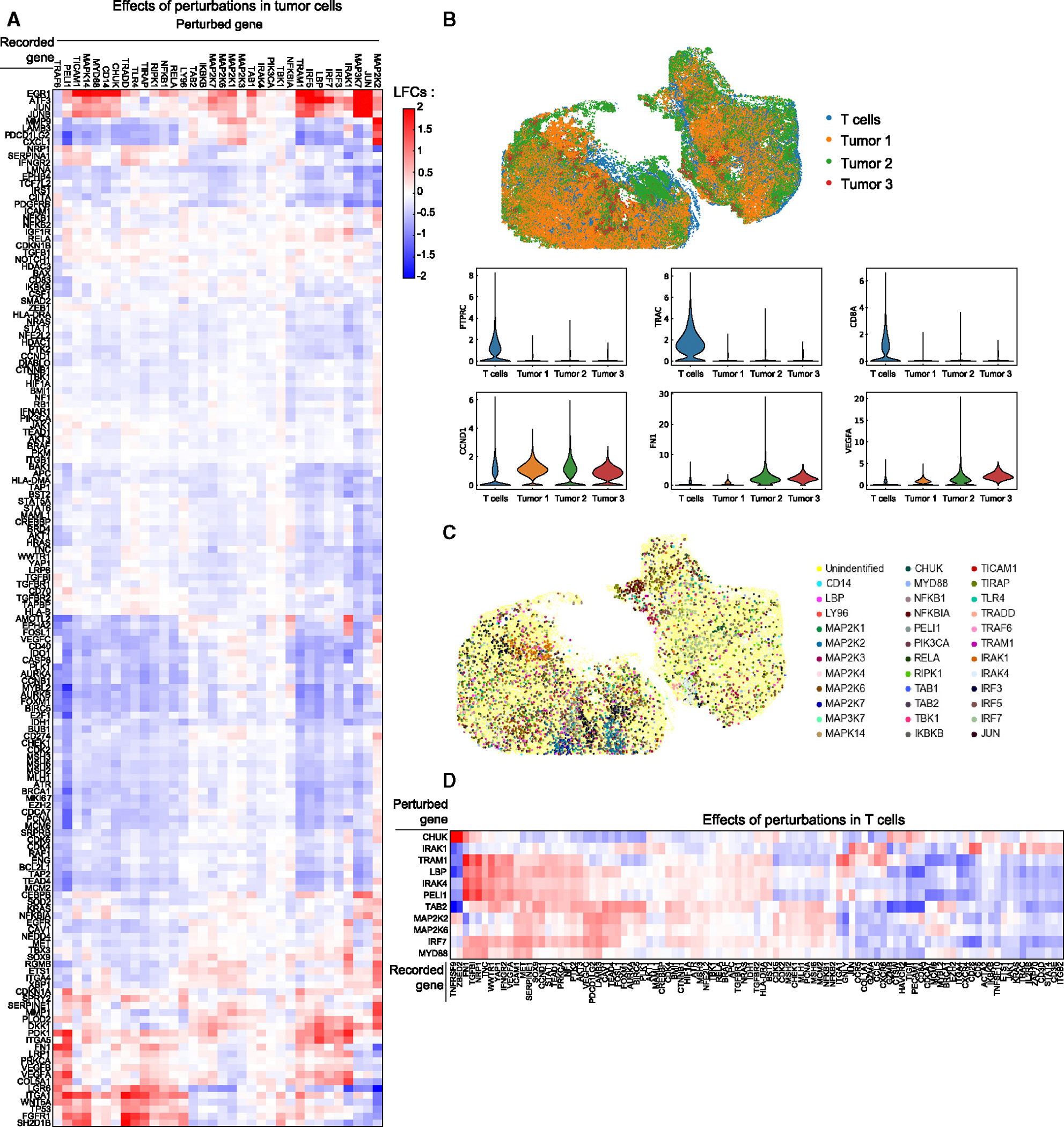
Perturb-FISH screen of NF-κB pathway in a tumor xenograft (A) Heatmap of intrinsic LFC effect sizes (color bar) of perturbations (x axis) on gene expression (y axis) in tumor cells. (B) Top, spatial map of cell-type identity in the tumor. Infiltrated T cells and three groups of tumor cells are identified using only their transcriptomes. Bottom, example violin plots for six genes that represent the signatures of the four identified clusters. (C) Spatial map of identified CRISPR-KO perturbations. (D) Heatmap of LFC effect sizes (color bar) of perturbations in tumor cells (y axis) on gene expression in T cells (y axis). See also Figure S5.

After analysis using FR-Perturb (STAR Methods), we recovered 2,022 significant cell-intrinsic effects from 32 perturbations that significantly affect the expression of at least 3 genes and 164 genes that are significantly affected by at least 1 perturbation (Figure 6A). We again verified that inferred effects were consistent between random subsets of the data (Pearson correlation: 79% for effects significant in either subset, 95% for effects significant in both) (Figures S5A and S5B). Providing further validation, many cell-intrinsic effects have been observed in prior studies in tumor cells (Table S1) or are consistent with known biology. For instance, perturbations to genes with known and shared functional roles—*MAP3K7* and *JUN*, which are both known to regulate proliferation among other processes—resulted in highly correlated downstream effects, including the upregulation of *ATF3* (LFC_MAP3K7_: 2.9, Q_MAP3K7_: 0.0007; LFC_JUN_: 1.9, Q_JUN_: 0.0007), *CEBPB* (LFC_MAP3K7_: 1.1, Q_MAP3K7_: 0.0007; LFC_JUN_: 1, Q_JUN_: 0.0007), *JUN* (LFC_MAP3K7_: 2.7, Q_MAP3K7_: 0.0007; LFC_JUN_: 2, Q_JUN_: 0.0007), and *JUNB* (LFC_MAP3K7_: 2.8, Q_MAP3K7_: 0.0007; LFC_JUN_: 2.3, Q_JUN_: 0.0007). Genes involved in migration and remodeling, such as *VEGFA*, *VEGFB*, *PDK1*, *FN1*, and *COL5A1*, are coherently upregulated in response to the KO of *NFKB1*, *TIRAP*, *TRAF6*, or *RIPK1*. Similarly, genes regulating DNA repair, such as *MSH6*, *MLH1*, *CDCA7*, *MSH2*, and *CASP8*, are coherently downregulated by the KOs of *TRADD*, *LBP*, *TRAM1*, and *IRF5*.

The spatial nature of Perturb-FISH data enables deeper analysis of gene regulatory networks. First, we observe coherent spatial localization of gene expression patterns (Figure 6B; STAR Methods). We locate human T cells as cells expressing high *PTPRC*, *CD2*, and *TRAC* and observe areas within the tumor with high variability in the degree of infiltration. Cancer cells are defined as cells expressing high *MKI67*, *CAV1*, and *CTNNB1* and present spatial variations in the expression of *VEGFC*, *FN1*, and *NRP1*. Unsurprisingly, mapping the perturbations also reveals the presence of clonal populations throughout the tumor tissue (Figure 6C). We next split the melanoma cells in two groups based on the presence of an immune cell in contact with the tumor cell (STAR Methods). Computing the effects of the perturbation within each of these subpopulations reveals a strong correlation of effects between conditions (Figures S5C and S5D) with three notable outliers. *TP53* is downregulated by *MAPK14 KO* in cells with an immune neighbor (LFC: −0.297, *Q*: 0.06) but upregulated in the absence of one (LFC: 1.24, *Q*: 0.06). The interactions of *TP53* and *MAPK14* have been previously characterized as they regulate autophagy, a process that allows cancer cells to resist drug and radiation therapies.^77^ *TP53* is also overexpressed in tumors lacking *MAPK14*, while high levels of *MAPK14* have been found in advanced stages of gastric cancer.^78^ The senescence marker *CDKN1A* is downregulated by the KOs of *IRAK1* (LFC: −0.61, *Q*: 0.008) or *NFKBIA* (LFC: −0.46, *q*: 0.07) in the presence of an immune neighbor but upregulated by these KOs in the absence of one (respectively LFC: 0.59, *Q*: 0.06 and LFC: 0.23, *Q*: 0.5). *CDKN1A* expression is known to be regulated by these members of the NF-κB pathway, resulting in cell senescence.^79,80^

Finally, we investigated how gene expression of T cells is affected by perturbations in neighboring cancer cells (Figure 6D). We found 276 significant intercellular tumor-immune effects, which remained correlated when splitting the data into 2 random halves to check for consistency (*p* = 0.7 for effects significant in either half, *p* = 0.99 for effects significant in both halves) (Figure S5E). The KOs of *IRAK1*, *LBP*, or TAB2 in the tumor cells had strongly correlated effects on T cell gene expression, each downregulating activation markers like *TNFRSF9* (LFC: −0.33 and *Q*: 0.08, LFC: −0.4 and *Q*: 0.02, LFC: −0.5 and *Q*: 0.006, respectively) and *ZBED2* (LFC: −0.24 and *Q*: 0.16, LFC: −0.31 and *Q*: 0.05, and LFC: −0.4 and *Q*: 0.02, respectively). *CD8A* and *TIGIT* were also affected in very similar ways by LBP-KO (LFC: −0.32, *Q*: 0.03 and LFC: −0.28, *Q*: 0.009, respectively) and TAB2-KO (LFC: −0.35, *Q*: 0.02 and LFC: −0.36, *Q*: 0.006, respectively). *CD8A*, *TIGIT*, and *TNFRSF9* are known to be coexpressed in T cells,^81^ and their simultaneous overexpression correlates with poor prognosis.^81^ Conversely, these same genes are overexpressed in T cells neighboring tumors cells with KOs of *CHUK* (LFC_TNFRSF9_: 0.6, Q_TNFRSF9_: 0.003; LFC_ZBED2_:0.5, Q_ZEBD2_: 0.003; LFC_CD8A_: 0.09, Q_CD8A_: 0.6; LFC_TIGIT_: 0.2, Q_TIGIT_: 0.07) and *MAP2K2* (LFC_TNFRSF9_: 0.13, Q_TNFRSF9_: 0.4; LFC_ZEBD2_: 0.09, Q_ZEBD2_: 0.5; LFC_CD8A_: 0.12, Q_CD8A_: 0.2; LFC_TIGIT_: 0.04, *Q*_TIGIT_: 0.6). *MAP2K2*-KO in tumor cells also results in significant overexpression of the immune checkpoint receptor ligand *PDCD1LG2* (LFC: 0.2, *Q*: 0.01) and downregulation of the marker of cytotoxicity *GNLY* (LFC: −0.29, *Q*: 0.03). Interestingly, mutations in the *MAP2K2* gene have been implicated in the failure of immune checkpoint inhibitor treatment in melanomas.^82–84^ Markers of naive T cells *CXCR4*, *CD5*, and *CD28* were downregulated in the proximity of *IRF7*-KO (LFC_CXCR4_: −0.25, Q_CXCR4_: 0.01; LFC_CD5_: −0.27, Q_CD5_: 0.003; LFC_CD28_: −0.3, Q_CD28_: 0.03) but upregulated in the proximity of *IRAK1*-KO (LFC_CXCR4_: 0.06, Q_CXCR4_: 0.6; LFC_CD5_: 0.26; Q_CD5_: 0.04; LFC_CD28_: 0.31, Q_CD28_: 0.06). *IRF7* upregulation was shown to promote expansion of T and B lymphocytes,^85^ with its silencing having opposite effects. Confirming their opposite impact on T cells, high *IRAK1* expression is associated with poor prognosis and reduced benefits from immunotherapies.^86^

Overall, this data demonstrated the applicability of Perturb-FISH to infer intrinsic and extrinsic effects from CRISPR perturbations in tissue and provides additional insights into the mechanisms by which tumor cells regulate T cell activation.

## DISCUSSION

In the present study, we developed and evaluated a protocol, Perturb-FISH, using all-optical experimental methods to study the genetic and molecular associations of spatial and functional biology at single-cell resolution. Our analysis demonstrates that Perturb-FISH results produce robust inference of intracellular perturbation effects with power at least as good as Perturb-seq, with the caveat (discussed below) that Perturb-FISH captures expression for only a targeted set of genes. We further present a series of results that cannot be found using conventional Perturb-seq, as they depend on the spatial context or functional profiling of each cell, and finally, we demonstrate the applicability of the method to infer the effects of genetic perturbation *in vivo*.

Intracellular effects in our first screen (macrophages responding to LPS stimulus) have been well characterized, but using Perturb-FISH allowed us to observe how these responses depend on spatial context. Notably, we found that several hallmark genes of the innate immune response are differentially regulated depending on local cell density, and we observed that expression in control cells can be affected by perturbations in neighboring cells. Such density-dependent and intercellular effects at a single-cell level likely have a profound effect on population-level immune response and the balance between pro- and anti-inflammatory factors, with further studies needed to clarify how the environment shapes cellular responses and how cellular activity (or perturbation) shapes local environment.

A critical advantage of Perturb-FISH is the ability to match the intracellular effects of genetic perturbations with a functional readout, such as live calcium imaging. We demonstrated the feasibility of the experiment by screening ASD risk genes in astrocytes and recording calcium activity in response to an ATP stimulation. Unlike the THP1 screen, relatively little is known about the role of the identified risk genes. By linking genetic perturbation with gene expression and functional profiles, we were able to generate hypotheses about several of these risk genes regarding their effects on the transcriptome and role in calcium activity, not only in the form of genes that are differentially expressed in cells of different phenotypes but also as targets whose silencing sets a particular phenotype for the cell. In particular, we find several targets that push the cells to favor one of the naturally occurring calcium phenotypes. This may be of special interest as previous works have reported hyper calcium activity in ASD patients and cells from ASD patients,^39^ which results in a disruption of neuronal activity by impairing the controlled development of a synapse network. When used in conjunction with functional profiling, Perturb-FISH is not just a screen of gene interactions but also a screen for gene function. Our data also show clusters of risk gene perturbations, suggesting they may act on common pathways in astrocytes in ASD. While astrocytes’ role in ASD was generally thought to be reactive to changes induced by neurons, recent evidence from larger-scale sequencing studies of primary tissue has shown ASD risk gene enrichment in glial cells.^38–40^ Further, our implementation of Perturb-FISH in tumor xenografts highlights how the method can be used to understand the mechanisms behind complex intercellular regulation of immune activation *in situ*.

With a guide barcoding strategy that mimics MERFISH, Perturb-FISH offers the same scalability in number of perturbed genes as MERFISH does in terms of recorded genes. For instance, as many as 140 gRNAs can be identified in 15 images (bits) with 5 rounds of imaging in 3 colors or as many as 316 gRNAs in 21 bits, or 550 in 25 bits. The number of genes that MERFISH records scales identically with the number of cycles, and the number of imaging cycles needed for Perturb-FISH is the sum of both. Furthermore, we have shown that CRISPR-KO and CRISPRi are both compatible with Perturb-FISH, but in principle, the method is compatible with any of the array of gRNA-based perturbations including base editing, CRISRP-a, CRISPR-off, and non-Cas9-based systems such as Cas12a or Cas13. While this work was in revision, two additional demonstrations of the feasibility of identifying gRNAs with a spatial readout by inserting a T7 promoter within the U6 promoter were published. The present work highlights the biological discovery possible when coupling this gRNA readout approach with functional phenotypes and the transcriptome, especially for the detection of intercellular effects, but these additional works add more evidence to the efficiency of the approach, as well as alternative sites where the T7 promoter can be inserted and a degree of optimization in the local sequence of the vector.^87,88^

The ability to interrogate the extracellular effects of cellular perturbations is a key step in understanding how they might generate function at the tissue scale. Using Perturb-FISH to decipher intercellular networks will require thinking differently about CRISPR screens. One might generally want to scale up the experiment to screen as many genes as possible; and Perturb-FISH can indeed achieve large numbers of perturbed genes, but looking at their effects on the neighbor cells requires finding enough occurrences of a particular cell pair (one control touching one perturbed cell). The frequency of such occurrences is the product of the proportion of controls and a specific perturbation. Therefore, if an experiment is designed to measure the extrinsic effects of guide perturbations, the relative proportion of control guides will directly affect the power of the screen (as will total number of cells, cell density, and number of perturbations), and hence such screens will likely need to include a much higher proportion of control guides than a typical Perturb-seq screen.

Overall, we believe Perturb-FISH advances functional genomics for the study of intracellular circuitry, while also enabling the study of the genetics of intercellular circuitry with rich molecular detail.

## Limitations of the study

A key limitation of our method is the focus on a targeted set of genes for transcriptomic readout, which limits the potential for *de novo* discovery. This limitation can be addressed in the future in several ways. First, while we have designed the chemistry around MERFISH binding and readout, we note that multiple hybridization-based chemistries are compatible with Perturb-FISH, including CosMx,^89^ STARmap,^90^ and Seq-FISH+.^91^ Notably, each of these has been shown to be compatible with higher plexes, up to 10,000 genes. Second, while we relied on commercial reagents to simplify development, we note that a fully home-brewed approach could decrease reagents costs dramatically (as much as 10-fold) and thus increase the number of genes profiled and perturbed. Finally, optical improvements maximizing the coverslip active area can substantially increase the number of cells profiled per dollar. Thus, we believe that Perturb-FISH is best thought of not as an alternative to scRNA-seq but as a separate tool used to profile phenotypes dependent on a cell’s native environment and guided by alternative experiments (e.g., our knowledge of the LPS response pathway or of ASD-involved genes). Furthermore, we note that the biological results are starting points highlighting Perturb-FISH’s utility, but they would still require additional investigation in more sophisticated model systems. Cell line results, such as those found in astrocytes, would need to be validated in either mouse brain or human organoids; and tumor xenograft results would need to be confirmed in more representative melanoma models. The strength of Perturb-FISH is that it permits initial investigation of large numbers of perturbed genes, which previously would have taken dozens to hundreds of parallel experiments.

## RESOURCE AVAILABILITY

### Lead contact

Further information and requests about reagents and other resources used in this work should be directed to and will be fulfilled by the lead contact, Samouil L. Farhi (sfarhi@broadinstitute.org).

### Materials availability

Plasmids generated in this study are available upon request to the lead contact and with a complete materials transfer. The plasmid used to insert gRNAs for Perturb-FISH experiments is available on Addgene (plasmid #220626).

### Data and code availability

- Tables of raw data, processed tables, and raw images are made available by SSPSYGENE at https://sspsygene.ucsc.edu/projects/. The raw data for the compressed sensing Perturb-seq screen is available on the NCBI website under accession number GSE221321.
- All original code has been deposited on GitHub (see key resources table) and is publicly available as of the date of publication.
- The protocol is available on protocols.io (https://doi.org/10.17504/protocols.io.6qpvr356bvmk/v1).

## STAR★METHODS

### EXPERIMENTAL MODEL AND STUDY PARTICIPANT DETAILS

#### Generating Cas9 expressing cell lines

THP1 cells from ATCC^®^ (TIB-202^™^) were maintained in RPMI (ATCC, RPMI-1640) with 10% FBS (ATCC^®^ 30–2020), and 0.05mM 2-mercaptoethanol, without antibiotics, at 37C. Cells were split to maintain a density between 500k cell/mL and 1.8M cells/mL. THP1s were infected with Cas9 virus by preparing 20 wells of a 96 well plate with 200k cells per well, with 50μL medium and 150μL virus (in medium) in 8 μg/mL polybrene. Cells were spinfected at 1000g for 90 min, then placed overnight in an incubator at 37C. The following morning medium was replaced with fresh maintenance medium and cells were left to recover for 2 days. Cells were then selected in medium containing 6μg/mL basticidin until control wells that received no virus were dead (8 days). Cells were then amplified for 20 days and stocks were frozen. We assessed Cas9 activity by infecting these cells (same protocol) with a virus containing both GFP and a guide against GFP. After puromycin selection, we used cytometry to quantify the number of cells that were not GFP positive (cells in which GFP was knocked out by Cas9 activity) and found 73% activity (Figure S4).

Astrocytes were differentiated from the UCSFi001-A line (human pluripotent stem cells from healthy male donor) expressing dCas9-KRAB following our previously published protocol.^55^ Specifically, on day 0, hPSCs were differentiated in N2 medium [500 mL DMEM/F12 (1:1) (Gibco, Cat # 11320–033), 5 mL Glutamax (Gibco, Cat # 35050–061), 7.5 mL Sucrose (20%, SIGMA, Cat # S0389), 5 mL N2 supplement B (StemCell Technologies, Cat # 07156)] supplemented with SB431542 (10 μM, Tocris, Cat # 1614), XAV939 (2 μM, Stemgent, Cat # 04–00046) and LDN-193189 (100 nM, Stemgent, Cat # 04–0074) along with doxycycline hyclate (2 μg.mL-1, Sigma, Cat # D9891) with Y27632 (5 mM, Stemgent, Cat # 04–0012), at 37C. Day 1 was a step-down of small molecules, where N2 medium was supplemented with SB431542 (5 μM), XAV939 (1 μM) and LDN-193189 (50 nM) with doxycycline hyclate (2 μg.mL-1) and Zeocin (1 μg.mL-1, Invitrogen, Cat # 46–059). On day 2, N2 medium was supplemented with doxycycline hyclate (2 μg.mL-1) and Zeocin (1 μg.mL-1). Starting on day 2 human induced neural progenitor-like cells were harvested with Accutase (Innovative Cell Technology, Inc., Cat # AT104–500) and re-plated at 15,000 cells.cm^−2^ in Astrocyte Medium (ScienCell, Cat # 1801) with Y27632 (5 mM) on geltrex coated plates.

#### Establishing PBMC-humanized mice and xenograft tumor model

NOD.Cg-Prkdcscid Il2rgtm1Wjl/SzJ (NSG, stock number 005557) mice were bred and housed at the Broad Institute’s animal facility following the institute’s animal care and use guidelines. PBMC engraftment and tumor challenge were performed under protocol number 0110–08-16–1, approved by the Broad Institute Institutional Animal Care and Use Committee.

Human peripheral blood mononuclear cells (PBMCs) were isolated from a Leukocyte Reduction System (LRS) cone (STEMCELL Technologies, Catalog #200–0093) using SepMate^™^−50 tubes (STEMCELL Technologies, Catalog #15450) and Ficoll^®^ Paque Plus (Cytiva 17–1440-02) density gradient centrifugation. The PBMCs (10 million cells per mouse) were injected intravenously into 8-week-old NSG mice and allowed 12 days for *in vivo* expansion prior to use.

A total of 5×10^5^ A375 cells expressing Cas9 were transduced with an NF-κB sgRNA library lentivirus at an infection rate of 30%. After 48 hours, transduced cells were selected with 2 μg/ml of puromycin for 10 days and scaled up for tumor challenge. The library cells were harvested, counted, washed twice with ice-cold Hanks’ Balanced Salt Solution (HBSS, Gibco), resuspended in HBSS, and mixed with 50% growth factor-reduced Matrigel (Corning). A total of 3.5 million tumor cells (200 μL) were injected subcutaneously (s.c.) into the right flank of each PBMC-engrafted NSG mouse.

Fourteen days post-tumor injection, tumor tissues were extracted, embedded in O.C.T., flash-frozen in liquid nitrogen, and stored at −80°C. One day prior to tumor extraction, PBMC engraftment rates were assessed by detecting human CD3+ CD45+ T cells in mouse peripheral blood (Figure S6A). Briefly, 20–30 μL of tail vein blood was collected in 5 mM EDTA-coated tubes, followed by the addition of 1 mL ACK lysing buffer (Gibco) to remove red blood cells. The cell pellet was washed twice with PBS/2% FBS buffer and incubated with 2 μg/mL of Brilliant Violet 421^™^ anti-human CD45 (clone 2D1, BioLegend) and FITC anti-human CD3 (clone OKT3, BioLegend) on ice for 30 minutes. After two washes with PBS/2% FBS, samples were analyzed using a Beckman CytoFLEX LX.

A375 cells were grown in DMEM (Gibco) + 10% FBS + antibiotics (50 U/mL penicillin, 50 μg/ml streptomycin), at 37C.

### METHOD DETAILS

#### Selecting genes to be measured with MERFISH

We used data from our matched Perturb-Seq^18^ screen to select an informative list of 130 genes to measure with MERFISH in our LPS response screen. Perturb-Seq data allowed us to establish modules of genes with correlated expression levels after LPS-stimulation. We selected a list of genes capturing major directions of variation (as determined by PCA), with a total expression across these genes below 2000 FPKM, and no individual gene above 30 FPKM which are conditions imposed by the use of MERFISH: high abundance creates crowding in the image and prevents decoding. In brief, we ran a principal component analysis on the raw counts matrix and scored every gene for how much of the variance of a principal component they captured. The list of genes was built by iteratively adding new genes. At each iteration, we simply cropped the original count matrix to estimate the count table we would get if we ran MERFISH with that list of genes. We then used a linear regression to fit this estimated data onto the PCA space, and calculated residuals and explained variance. The principal component with the lowest explained variance received 2 additional genes, which were chosen for being the most correlated with the residual.

For the ASD screen, we used a list of risk genes published by the SFARI consortium (50 genes), to which we added genes from a published work for Sattertrom^64^ and control genes known to regulate ATP signaling of calcium activity, for a total of 127 target genes. Since little is known about most of the 127 genes selected for perturbation, we chose to record the same list of genes with MERFISH. In addition, we selected 358 genes that are differentially expressed in astrocytes between healthy patients and people with ASD,^34,35^ totaling 485 genes.

For the screen in tumor xenografts, we re-used the guide library targeting the NFκB pathway, and recorded genes using the pre-designed immuno-oncology panel (10400150) from VIZGEN.

#### Virus preparation

Viruses were prepared using a previously published protocol (https://portals.broadinstitute.org/gpp/public/dir/download?dirpath=protocols/production&filename=TRC%20shRNA%20sgRNA%20ORF%20Low%20Throughput%20Viral%20Production%20201506.pdfhttps://portals.broadinstitute.org/gpp/public/dir/download?dirpath=protocols/production&filename=TRC%20shRNA%20sgRNA%20ORF%20Low%20Throughput%20Viral%20Production%20201506.pdf). Cas9 virus was concentrated 10 times by centrifugation through a 100kD purification column.

#### Vector design and cloning guide libraries

LentiGuide-Puro was a gift from Feng Zhang (Addgene plasmid # 52963). The vector was modified to replace the sequence of the gRNA scaffold with the optimized sequence from Hill et al. 67, add a T7 between the end of the U6 and the cut site of BsmbI20, we also removed the T7 region that is part of the LentiGuide-Puro to only keep one. The final plasmid was purchased from Genscript and is available on addgene.

We designed libraries containing 2 guide RNAs per target gene. Guides were designed using the CRISPick tool from the Broad institute 30 base pair cloning Gibson arms were added on either side during synthesis. These are complementary to the flanking regions on the vector plasmid and are therefore the T7 promoter on the 5’ side, and part of the optimized scaffold on the 3’ side. We manually replaced guides (with the next best guide from CRISPick^3,92^) when stitching these regions resulted in sequences of more than 3 consecutive Gs as we found these impaired proper amplification before cloning and presumably also during T7 transcription. Guide libraries were ordered as oPools from IDT (libraries were ordered several times within one single oPool to minimize variations in guide frequencies). The library was PCR amplified and assembled using a Gibson assembly. The transformed plasmids were electroporated into competent bacteria (electroporation results in more uniform libraries than heat shock) and maxi prepped. 2 non targeting guides (not matching anywhere in the genome), and 2 safe targeting guides (matching a non-coding region) were cloned separately and added at the end to represent 30% of the final library. The final library was sequenced using a MiSeq to verify guide distribution. Briefly, two PCRs were used to amplify the guide region, and first add the proper read1 and read2 priming regions then the P5 and P7 regions. We spiked 30% PhiX into the library before loading on the MiSeq to compensate the relative low variability of this library.

#### Infecting cells with guides libraries

Guides were inserted in Cas9 expressing THP1 cells by spinfecting 40 wells of a 96 well plate with 200k cells per well, with 2μL of virus and 8μg/mL polybrene for one hour at 800g. Spinfection was done in the evening, and the following morning the cells were washed and placed in normal medium at 800k cells /mL. After one day rest, cells were selected in puromycin 4μg/mL until a control well was fully dead (2 to 3 days). The amount of guide virus to use was established by infecting cells with serial amounts of virus (from 0.5μL to 50μL), selecting cells with 4μg/mL puromycin for 3 days. After selection, numbers of cells were compared to a well that was spun with polybrene but no virus to find the amount of virus to use to obtain 25% survival, which corresponds to a MOI (multiplicity of infection) of 0.25.

Astrocytes were infected with lentivirus on day 24, using the same infection protocol as for the THP1s but with 50,000 cells and 3.67μL virus per well of a 96 well plate, selected with 4μg/mL puromycin and were ready for imaging on day 30.

#### Validation of knockout efficiency

Cas9 efficiency was validated by infecting Cas9-expressing THP1s with a vector encoding for both GFP and a guide against GFP (under “normal” U6 promoter). Knockout efficiency (73%) was evaluated by FACS (Figure S6B). Separately, we infected HELAs expressing Cas9 under a Dox-inducible promoter with GFP, and 2 gRNA against GFP under a U6T7 promoter. We validate the efficiency of the knockout by imaging (Figure S6C) and by flow cytometry (Figure S6D). In a third experiment, we inserted single guide perturbations targeting CD14 in THP1s, selected the cells in puromycin for 3 days. 6 days after infection, we stained the cells for CD14. Cells were detected using the MATLAB function imfindcircles on brightfield images (Figure S6E), positive cells were identified by thresholding the intensity at 400 on fluorescence images. As all THP1s are not expected to express CD14,^93^ we compare the percentage of CD14 positive cells in KO cells to the control cells and reach a knockout efficiency of 79% with 7.6 +/− 2.6% cells being CD14+ in the control, versus 1.9+/−0.13% in the KO (Table S3).

#### THP1 plating and stimulation

THP1 cells were plated in a polyornithine/laminin coated 6 well plate, at 1 million cells per well, in medium containing 20ng/mL phorbol-12-myristate-13-acetate (PMA). After 24 hours, medium was replaced with fresh medium without PMA. 2 hours later, cells were lifted with Trypsin and replated on polyornithine/laminin glass. The glass used was the circular coverslip compatible with MERFISH, provided by Vizgen. We used removable chambers from IBIDI to restrict the plating area and only plate the number of cells that could be later imaged. 42k cells were plated in 0.22 cm^2^ for the negative control, and 100,000 cells were seeded in 0.56 cm^2^ chambers, on the same glass slide, to generate wells that would be later stimulated with LPS. One day later, medium was replaced with either fresh medium or medium containing 100ng/mL LPS. Cells were then incubated for 3 hours prior to fixation.

#### Guide RNA codebook design

Following the approach used in MERFISH, we use a Hamming weight 4 Hamming distance 4 codebook to encode the identities of our guide RNAs. Every guide is encoded as a binary barcode that contains exactly 4 “ones” and any 2 barcodes from the codebook are different on a least 4 bits. This approach allows for single bit error correction as a barcode that contains an error has only one bit different from its actual matching code, and at least 3 bits difference with all others. Barcodes that contain 2 errors are discarded as they could match 2 possible barcodes. We find that certain combinations of ones and zeros are particularly sensitive to false positive detection, or dropout and should be avoided: all sequences that would result in a guide always lighting up in the same color, or in a guide being “on” in all 3 images in one single round should be avoided.

#### Perturb-FISH encoding probes

After T7 transcription, each cell contains multiple copies of RNA at the site of genomic insertion. These RNA molecules started with the gRNA sequence followed by the optimized guide scaffold used in our vector. We targeted these regions with encoding probes that contained a 30-base pair annealing region matching the gRNA and the first 10 base pairs (bp) of the optimized scaffold. This allowed a hybridization region of 30 base pairs, which is also the length used in the MERFISH probes, therefore allowing similar sensitivities to temperatures and formamide concentrations. Each hybridization region was flanked with two 20 bp readout regions on either side that were complementary to the 4 readout probes used to encode the 4 bits that characterized each gRNA.

#### Perturb-FISH readout probes

For the THP1 experiment, all readouts (for guides and genes) were purchased as a kit from Vizgen. For the astrocytes, fluorescent readout probes were acquired from Eurofins with sequences matching those published by C. Xia et al.^70^ (Table S1).

These oligos are tagged with a fluorophore attached to the 5’ side with a disulfide bond. The readout buffer was made of 10mL 50% ethylene carbonate 5mL 20X SSC, 34 mL RNAse free water, 1mL 25% Triton-X 100, 200uL tween-20 and 50μL murine RNAseI as used before by others.^94^

#### Perturb-FISH protocol

Cells were washed twice in PBS, then fixed on ice in methanol/acetic acid at a 3:1 ratio as done by A. Askary et al.^17^ As the authors of this work note, it is important not to fix with PFA as this blocks T7 transcription. Cells were washed in PBS by doing exchanges to prevent the sample from completely drying. Coverslips were placed in a 60cm petri dish, filled with PBS, and the IBIDI chambers were then peeled from the glass, allowing all conditions to receive the same later incubations. T7 transcription mix was prepared following manufacturer (Thermofisher, AM1334) instructions: 8μL of each dNTP was mixed with 8uL 10x reaction buffer, 8μL T7 enzyme, and 32μL nuclease free water. The liquid covering the coverslip was then removed, a drop of the transcription mix was pipetted on top of the areas with cells and covered with a square of parafilm to spread the drop and prevent evaporation. Coverslips were incubated at 37C for 6 hours, then fixed without washing, in 4% PFA, for 15 minutes, then washed 3 times 5 minutes in PBS and stored overnight in 70% ethanol. Coverslips then underwent normal MERFISH preparation: samples were washed in formamide wash (5mL 20X SSC, 15 mL 100% deionized formamide, 30mL nuclease free water) at 37C for 30 minutes. An encoding buffer was then prepared by mixing the MERFISH encoding library provided by VIZGEN with probes targeting the guide RNA regions from a stock at 300X concentration to reach a final concentration of 1nM per probe, plus 4nM of 5’acrydite-modified guide anchors GCGCCAAAGTGGATCTCTGCTGTCCCTGTAA, and GGATGAATACTGCCATTTGTCTCAAGATCTA that anneal to the T7 transcribed RNA downstream of the scaffold and will later anchor the transcripts to the acrylamide gel. The coverslips were placed overnight at 37C in a cell culture incubator. The sample was then washed twice for 30 minutes in formamide wash buffer at 47C. Finally, the sample was washed 3 times in 2X SSC for 2 minutes each, then incubated for 10 minutes in 5mL 2X SSC containing 1μL of 5000X fiducial beads solution (Polysciences, #17149–10). The sample was then embedded in a polyacrylamide gel, then cleared for one day at 37C following the protocol used in MERFISH. When necessary, the samples were stored in 2X SSC containing 1μL/mL murine RNAseI. After clearing, samples were bleached in 2X SSC for up to 12 hours under a blue LED to further reduce background noise.

In the case of the xenograft experiments, we used the same protocol for gRNA amplification, but the sample was then prepared using VIZGEN’s FFPE protocol (10400150). Briefly, this approach functionalizes the RNA to prepare for their immobilization in the acrylamide gel, then the sample is gel embeded, cleared, and only then incubated in the encoding probes against both the transcriptome and the perturbations. This resulted in improved RNA quality, which was critical for the implementation of Perturb-FISH in tissues.

#### Sample Imaging

Cells were stained with DAPI 1/1000 for 5 minutes and imaged on an alpha version of Vizgen’s MERSCOPE. Images dedicated to the detection of guide RNAs were acquired first. Briefly, 5 automated cycles of imaging were run to collect all 15 bits encoding the guide library used on THP1s. Each cycle started with 15 minutes incubation of the next readout buffer (containing 3 readout probes with dyes at 750nm, 647nm, and 565nm). The samples were then washed for 15 minutes in wash buffer (5mL 20X SSC, 10mL 50% ethylene carbonate, 35mL RNAse free water). The wash buffer was then replaced with imaging buffer made of 15mL 2X SSC, 1.66mL Trolox quinone at 500μM [Mix 395mg Trolox in 15.8mL methanol. Dilute 0.4mL of that solution in 20mL 2X SSC. Place in UV crosslinking oven and bake for 1 hour at 254 nm. Measure absorbance at 255. The concentration in μM is (abs(255nm) – 0.8)/11200*1000000. Dilute in 2X SSC to 500 μM], 33μL protocatechuic acid (2.5M), 83μL Trolox 10% in methanol, 33μL recombinant protocatechuate 3,4-dioxygenase, 33μL RNAseI, and NaOH to adjust pH to 7. Each imaging round finished with a 15-minute incubation in extinguishing buffer (2X SSC with 50mM TCEP) followed by a 10-minute rinsing step in 2X SSC with RNAseI. After acquisition of the images of the guide RNAs, the transcriptome was imaged using reagents provided by VIZGEN. It is critical that buffers do not stay at room temperature for too long as their degradation impairs the detection of transcripts/guides, and we therefore ensured that the automated fluidics system was only loaded with the buffers required for the next 12 hours of imaging. Buffers were added to the fluidics system as imaging progressed as a complete Perturb-FISH run can easily require 5 days of imaging.

#### Decoding guide identity

Raw images were registered using previously published algorithms used for MERFISH image analysis71. Image preprocessing was performed similarly to Lu et al.^72^ Images were divided by images of the background in each channel to correct for uneven illumination across field of views. Spots were then enhanced with a gaussian filter of size 2 using MATLAB’s imgaussfilt. Background was computed as the same image filtered with a gaussian filter of size 12 and subtracted. Image stacks of 7 Z-levels were then max-projected. These images were then binarized using a simple threshold and filtered to remove spots smaller than 7 pixels or bigger than 450 or with an eccentricity over 0.8. For each field of view, images from all channels were then max-projected to find the locations of putative gRNAs. For each of these putative guides, a centroid position was determined, and the sequence of images that contained a binary object within a radius of 5 pixel of this centroid was compared to the codebook of the guide library. The use of a Hamming weight 4 Hamming distance 413,14 codebook allows identification of all guides that fluoresce in 3, 4, or 5 images. We then used the standard deviation of spot intensity and their average fluorescence across image rounds to generate scatter plots (Figure S6F). On these plots, 2 clouds of points were easily identifiable, one of them is enriched in blank barcodes that do not actually match any gRNA from the library. We therefore considered this cloud as misidentified gRNAs and filtered these false gRNAs out of our analysis. In the THP1 dataset, out of 14,864 decoded spots, 22 matched blank IDs before this filtering, which implies an error rate of 2.74%. After filtering, 5 spots matching a blank barcode remain out of 13,216 remaining, which corresponds to a 0.7% error rate.

mRNA images were decoded using MERlin with default parameters. (https://github.com/emanuega/MERlin, https://zenodo.org/records/3758540)

After all filtering and QC steps, the THP1 data contains a median number of 150 cells per perturbation, with 2,184 control cells. The astrocyte data contains a median number of cells per perturbation of 86, with 2,634 control cells (non- and safe-targeting guides). The tumor data has a median number of cells per perturbations of 125, with 4,444 control cells.

#### Cell segmentation

THP1 cells were segmented using a watershed algorithm. Nuclei were segmented on each field of view, and a binary mask of nuclei centroids was then created to use as seeds for the watershed algorithm. To generate basins, we used a map of all transcripts decoded by running Merlin on our MERFISH images, then used a gaussian filter to blur them out, resulting in a mRNA density map of the sample.

Astrocyte nuclei were segmented using a binary mask of DAPI signal, then morphologically dilated this mask. These images (acquired at 60X) were downscaled to match the dimension of the calcium images (2X) and recognizable features in the dish were manually identified and used to compute an affine geometrical transformation using MATLAB’s estimateGeometricTransform function to realign images across modalities. Images were then translated using imwarp_same.

Tumor images were segmented using a similar approach to the one used for the astrocytes. Perturbations and transcriptome images were registered using the same approach used for the astrocytes.

#### Generating count tables

Count tables were assembled using previously built masks and matrices of decoded transcripts: for each object of the mask, the number of transcripts for each gene was summed. Design matrices containing the identity of the perturbation received by each cell were assembled in the same way. THP1 expression tables were filtered to remove cells with less than 70 or more than 2300 counts. Expression tables for the astrocytes were filtered to keep cells with counts between 200 and 1900, and genes with at least 0.5 counts per cell. In the absence of prior knowledge of the system, we applied stringent filters on the expression data. We identified lowly expressed genes and removed expression level information for these genes from the dataset leaving 127 perturbations and 277 measured genes. Finally, for the tumor data, genes with less than 0.3 counts per cell on average were removed to build 2 lists of genes: genes expressed in tumor cells, and genes expressed in infiltrated lymphocytes. Tumor cells were filtered to keep cells with a total count between 40 and 800 and a surface area between 2,000 and 30,000 pixels.

#### Neighbor detection

To identify the neighbors of a given THP1 cell, we dilated its mask by 50 pixels (5.4μm) in all directions in the MERFISH data, or by 10 pixels (6.2μm) in the RNAscope data. Any cell that overlapped with this dilated mask was identified as a neighbor. Cells with 2 or fewer neighbors were called “low density” cells, while cells with 3 or more neighbors were called “high density” cells.

In the tumor, neighbors were identified with a similar algorithm, with the dilation set to 30 pixels.

#### Cell type labeling in the tumor

Cell typing was performed using scanpy on log-transformed raw counts matrices. Leiden clusters were identified using default parameters and labeled as clusters of tumor cells or infiltrated T-cells based on their transcriptomes. Cluster 0 had very low counts and was discarded as putative mouse cells for which our human probes had low affinity, and cluster 7 was discarded as a cluster of doublet cells.

#### qPCR validation of effects

THP1 cells were transduced with a modified gRNA vector containing both puromycin resistance and GFP. After selection, cells were mixed at ratios of 14% perturbed cells with 86% non-perturbed controls (received a non-targeting guide) and seeded in 6 well plates with 20ng/mL PMA for differentiation. After a day, cells were re-seeded in fresh medium in a 48 well plate at different densities: low density: 25,000 cells/cm2, high density: 216,000 cells/cm2. Cells were stimulated for three hours in LPS at 100ng/mL, then detached and sorted perturb and control cells for each density by FACS based on GFP expression and extracted their RNA using a miniprep kit (NEB, T2010S), performed reverse transcription (NEB, E3010L) and ran qPCR (NEB, M3003L) in triplicates. Actin-B was used as a reference for transcript abundance.

#### RNAscope data generation

For effect validation using RNAscope data, cells were transduced with a modified gRNA vector containing both puromycin resistance and GFP. Cells were prepared following the same protocol as for the qPCR and seeded in 96 well plates at the same density as before. Samples were stained following the manufacturer’s protocol16 and imaged at 10X on a Nikon Ti2-E microscope. GFP signal is used to identify cells that received a perturbation.

#### Calcium imaging

Astrocytes were grown on glass coverslips (VIZGEN 10500001), in chambers made of PDMS (poly dimethyl sulfoxide). Cells were loaded for 30 minutes with 2uM Fura-4AM dye at 37 °C, then washed and imaged in recording solution (125mM NaCl, 2.5mM KCl, 15mM HEPES, 30mM glucose, 1mM MgCl2, 3mM CaCl2 in water, pH 7.3, mOsm 305) as detailed in M Berryer et al.^5^ Time lapse videos were acquired at 2X on a Nikon Ti2_E microscope equipped with a Photometrics Iris 9 camera at 2 Hz images per second under 488 nm illumination provided by a Lumencor Celesta, emission signal was acquired through a Chroma ET560/40x filter. Cells were stimulated by adding one volume of recording solution with 200μL of ATP at 500μM to the one volume already in the well, for a final concentration of 250μM. Cells were then fixed and carried through to the Perturb-FISH protocol.

For each cell, signal intensity across time was computed as the mean value of all pixels in the cell, using the aligned cell mask from MERFISH. The base level of each trace was subtracted, and peaks were detected using a findpeak algorithm from MATLAB, with thresholds set to 15 for the minimal height, 6 for the prominence (local height) and 15 for the space between peaks. Traces were not further normalized because the absolute height of the calcium response carried biological information.

For parametrization of this data, we used the number of peaks, the local peak height (prominence), the size of the first step (defined as the difference between the maximum value of calcium intensity in the 5th to 15th frames and the minimal value between the 1st and 10th frames), the first response time (i.e., the delay before detection of the first peak, set to 200 – the duration of the analyzed video – when no peak was detected), the average peak delay, the area under the curve, and a binary parameter set to 1 if at least a transient was detected, 0 otherwise. These numbers were then Z-score normalized, and k-means clustering was used to cluster the cells into 6 groups of different signature calcium activity.

### QUANTIFICATION AND STATISTICAL ANALYSIS

#### Determining perturbation effect sizes

The effects of perturbations in THP1s were determined using our published tool FR-Perturb,^18^ using a rank of 34, lambda1 and lambda2 of 0, and log_exp_baseline of 2.7 and the total number of transcripts per cell as a covariate. P-values were evaluated with 10000 permutations of the perturbation assignment and corrected for multiple hypothesis testing using the Benjamini-Hochberg procedure. The script was slightly modified to remove the normalization to 10,000 counts per cell that is traditionally performed on droplet-based data. These parameters were determined by screening a range of values and keeping the ones that resulted in the highest correlation between effects computed on two random halves of the data. FR-Perturb outputs two tables: one contains the effects of the perturbations expressed as LFC, the other contains q values for significance analysis. Significant effects are here defined as effects with associated q value below 0.1.

In the part of the study in which we consider density, the population of cells was first divided depending on the number of neighbors of each cell. Low density was defined as 2 or fewer neighbors, and high density as 3 or more.

In the case of astrocytes, effects of perturbations were determined using our published tool FR-Perturb, using a rank of 114, lambda1 of 0 and lambda2 of 20, and log_exp_baseline of 5.5 and the table of transcript counts was normalized to 10,000 counts per cell. P-values were evaluated with 10,000 permutations of the perturbation assignment and corrected for multiple hypothesis testing using the Benjamini-Hochberg procedure.

In the case of the tumor, rank was set to 26, lambda1 to 0.6 and lambda2 to 2, with the same approach to determining significance on tables of raw transcript counts, with total counts as a covariate. When looking at perturbation effects inside T-cells, rank was set to 10, lambda1 to 0 and lambda2 to 0.5, on tables of raw transcript counts, with total counts as a covariate. When defining control cells, we used all T-cells that had only neighbors without a known perturbation other than control guides.

Log-fold-change (LFC) values all use natural log, all correlations are Pearson’s correlation.

Additionally, in some cases FR-Perturb may return many small but apparently significant effects. We observed this in our astrocyte dataset, with significant effects (q<0.1) having an average absolute magnitude of 0.05. This is partly explained by the use of sparsity-promoting penalties in FR-Perturb that result in coefficient shrinkage.^95,96^ To see this, we computed the same set of effects with a “naïve” approach that does not involve shrinkage. In the naïve approach, we compute the log-fold changes as the log of the ratio of the average gene expression profile of all cells with a given gene knock down to the average expression profile in control cells. Non-zero effects learned with this approach are 77% correlated with effects from FR-Perturb, and, as expected, are overall larger in magnitude (average effect size of 0.51 from naïve effects vs 0.063 from FR-Perturb). To assess reproducibility with the naïve approach, we computed LFCs on 2 randomly selected halves on the data and observed that significant effects are 67% correlated (in comparison with 94% correlation when using FR-Perturb on each half), and respectively 71% and 66% correlated with effects computed with FR-Perturb on the same half of the data (Figure S4C). Thus, although FR-Perturb estimates in our astrocyte data are smaller, they are comparable in relative effect size with the naïve approach, while also being substantially more reproducible. Overall, FR-Perturb allows higher confidence in recovered downstream targets of a perturbed gene, relative effect sizes, and correlation structure of effects even if the global scale of LFCs is low.

#### Astrocyte differential calcium profile and expression analysis

We used the distribution of control cells across calcium trace clusters to establish an expected distribution of cells between clusters. For perturbation to a target “A” we calculate enrichment or depletion in a cluster “B” as the log-ratio of the proportion of “A” cells falling in cluster “B” with the expected frequency based on the control distribution. For statistical analysis, we used a hypergeometric test on the raw values of guide abundance, where the p-value for the enrichment of guide A in cluster B is 1-hygecdf(x-1,M,K,N) with hygecdf MATLAB’s hypergeometric cumulative distribution function, x the number of cells with guide A in cluster B, M the total number of cells, K the number of cells in cluster B, N the total number of cells with guide A.

Gene expression signatures for each cluster were computed as the mean of the Z-scored expression of a gene for all cells in a cluster. Significance was established using an ANOVA test on raw expression levels. Clusters are characterized by different total detected RNA abundance levels (sum of all detected transcripts), with a difference of up to 11% fewer total counts in cells with a small peak compared to cells with large early transients (810 +/− 402 versus 898 +/− 409 detected transcripts, p=1.6.10–11; Figure S4F). In this analysis of expression signature in each phenotypic cluster, counts are normalized to total counts per cell. Again, we find that cells with large peaks express lower levels of our control gene *ITPR3* (z-scored expression: −0.8).

## Supplementary Material

Table S3

Table S1

Table S2

1

SUPPLEMENTAL INFORMATION

Supplemental information can be found online at https://doi.org/10.1016/j.cell.2025.02.012.

## Figures and Tables

**KEY RESOURCES TABLE:** 

REAGENT or RESOURCE	SOURCE	IDENTIFIER

Antibodies		

AF488-anti TLR4	Invitrogen	53-9917-42, lot2562921; RRID: AB_1963634
AF488-nti CD14	Invitrogen	53-0149-42, lot 2869688; RRID: AB_2744748
Anti IRAKI	Invitrogen	MA5-15783, lotzd4273851; RRID: AB_11154024
AF488-anti mouse	Invitrogen	A32723, lot yl386289; RRID: AB_2633275
BV421^™^ anti-human CD45, clone 2D1	Biolegend	Cat# 368522; RRID: AB_2687375
FITC anti-human CD3, clone OKT3	Biolegend	Cat# 317306; RRID: AB_571907

Bacterial and virus strains		

Stbl3 bacteria	Invitrogen	C7373-03

Biological Samples		

LRS Cone	Stemcell technology	Catalog # 200-0093

Chemicals, peptides, and recombinant proteins		

N2 supplement B	StemCell Technologies	07156
SB431542	Stemgent	1614
XAV939	Stemgent	04-00046
LDN-193189	Stemgent	04-0074
Y27632	Stemgent	04-0012
TransIT-LT1	Mirus	MIR 2304
polybrene	Millipore sigma	TR-1003-G
puromycine	Gibco	A11138-03
blasticidin	Gibco	A11139-03
lps	Sigma Aldrich	L4516-1MG
phorbol-12-myristate-13-acetate	Sigma Aldrich	P8139-1MG
Megascript T7 transcription kit	Thermofisher	AM1334
ethylene carbonate	Sigma aldrich	E26258-500g
fiducial beads	Polysciences	17149- 10
deionized formamide	J.T.Baker	4028-01
40% acrylamide/bis-acrylamide 19:1	Bio-Rad	1610144
Trolox	Millipore sigma	238813-5g
protocatechuic acid	Sigma Aldrich	37580
recombinant protocatechuate 3,4-dioxygenase	Oriental yeast co, ltd	46852004
TCEP	Thermo scientific	77720
ammonium persulfate	Sigma aldrich	09913-100mg
yeast tRNA	Invitrogen	Am7119
dextran sulfate, 50% solution	Millipore sigma	S4030
acetic acid, glacial	J.T. Baker	64-19-7
N,N,N,N'- Tetramethyl-ethylenediamine (TEMED)	Sigma aldrich	T9281-50mL
proteinase K	NEB	P8107s
RNAse inhibitor, murine	NEB	M0314L
Proteinase K	NEB	T2010S
RNA miniprep kit	Monarch	T2010s
Fura-4AM	Thermofisher scientific	F14201
Matrigel (Growth Factor Reduced)	Corning	cat # 356239
Ficoll^®^ Paque Plus	Cytiva	cat# 17-1440-02
ACK lysing buffer	Gibco	cat # A1049201

Critical commercial assays		

RNA scope kit	Advanced cell diagnostics	310091,322000, 323110,322381
MERFISH kitTHPIs	Vizgen	VZG109
MERFISH kit astrocytes	Vizgen	CP0710
MERFISH IO kit	vizgen	10400150

Deposited data		

Tables of raw and processed Perturb-FISH data	SSPSYGENE	https://sspsygene.ucsc.edu/projects/
Compressed perturb-seq data	NCBI	GSE221321
Raw images	SSPSYGENE	https://sspsygene.ucsc.edu/projects/

Experimental models: Cell lines		

THP1 (male)	ATCC	TIB-202^™^
Hek (female)	ATCC	CRL-1573
A375 (female)	ATCC	CRL-1619
iPSC (male)	NIH_M. Ward lab	UCSFi001-A
HELAS	ATCC	CCL-2^™^

Experimental models: Organisms/strains		

Mouse NSG	Jackson Lab, in house	NOD.Cg-Prkdcscid Il2rgtm1Wjl/SzJ, Stock No: 005557

Oligonucleotides		

Guide library THP1 and tumor xenograft	This paper	See Table S2
Guide library astrocytes	This paper	See Table S2
Encoding library THP1 and tumor xenograft	This paper	See Table S2
Encoding library astrocytes	This paper	See Table S2
Fluorescent readouts	This paper	See Table S2
Anchor 1: /5Acryd/ GCGCCAAAGTGGATCTCTGCTGTCCCTGTAA	IDT	N/A
Anchor 2: /5Acryd/ GGATGAATACTGCCATTTGTCTCAAGATCTA	IDT	N/A
Guide 1 against GFP: GAGCTGGACGGCGACGTAAA	IDT	N/A
Guide 2 against GFP: CTCGTGACCACCCTGACCTA	IDT	N/A

Recombinant DNA		

Pmd.2	addgene	12259
Pspax2	addgene	12260
LentiGuide-Puro	addgene	52963
Perturb-FISH plasmid	addgene	220626

Software and algorithms		

Code to analyze Perturb-FISH data	github	https://github.com/lbinan/Perturb-FISH
FR-Perturb	github	https://github.com/douglasyao/FR-Perturb
FlowJo v10.10	FlowJo, LLC	https://www.flowjo.com

Other		

MERFISH coverslips	VIZGEN	10500001
IBIDI cell culture removable chambers	IBIDI	81201,250210

## Highlights

- Perturb-FISH combines spatial transcriptomics and readout of CRISPR perturbation
- We recover the effects of genetic perturbation on the transcriptome of single cells
- We find specific genetic networks related to cell neighbors in tissue
- We connect genetic networks and time-resolved imaging phenotypes after perturbation
