# Supplementary material for: Simultaneous CRISPR screening and spatial transcriptomics reveal intracellular, intercellular, and functional transcriptional circuits
Source: Cell. Author manuscript; Available in PMC 2025 Jun 4. (PMC12135205; doi:10.1016/j.cell.2025.02.012)
Supplement: Table S3 [file NIHMS2080922-supplement-Table_S3.pdf]

	image	positive	total	Percentage of CD14+ cells
control	1	59	830	7.108433735
control	2	30	1002	2.994011976
control	3	72	666	10.81081081
control	4	73	777	9.395109395
control	5	70	888	7.882882883
			average	7.63824976
			st dev	2.648063231
KO	1	20	976	2.049180328
KO	2	12	645	1.860465116
KO	3	18	1049	1.715919924
			average	1.875188456
			st dev	0.13645074
			p value	0.017207834
