# Supplementary material for: Simultaneous CRISPR screening and spatial transcriptomics reveal intracellular, intercellular, and functional transcriptional circuits
Source: Cell. Author manuscript; Available in PMC 2025 Jun 4. (PMC12135205; doi:10.1016/j.cell.2025.02.012)
Supplement: Table S1 [file NIHMS2080922-supplement-Table_S1.pdf]

Cell, Volume *188*

Supplemental information

Simultaneous CRISPR screening and spatial transcriptomics reveal intracellular, intercellular, and functional transcriptional circuits

Loïc Binan, Aiping Jiang, Serwah A. Danquah, Vera Valakh, Brooke Simonton, Jon Bezney, Robert T. Manguso, Kathleen B. Yates, Ralda Nehme, Brian Cleary, and Samouil L. Farhi

Supplementary Table 1. Table of effects validated in the literature, related to figure 6.

Knockout gene	Affected gene	LFC	Q value	Refence validating effect
TRAF6	JUN	-0.32	0.004	[1]
TRAF6	VEGFA	1	0.003	[2]
NFKBIA	RELA	0.25	0.002	[3]
NFKBIA	MMP1	-0.34	0.008	[4]
RELA	CXCL2	-0.32	0.004	[4]
RELA	ICAM1	-0.31	0.004	[3]
MAP3K7	YAP1	-0.7	0.0007	[5]
MAP3K7	EGR1	7	0.0007	[6]
MAP2K2	EGFR	0.66	0.0007	[6]
JUN	YAP1	-0.41	0.0007	[7]
IRF7	IDO1	-0.7	0.0007	[8]
MAPK14	TP53	0.725	0.1	[9]

1. Li, J., Liu, N., Tang, L., Yan, B., Chen, X., Zhang, J., and Peng, C. (2020). The relationship between TRAF6 and tumors. Cancer Cell Int *20*, 429. 10.1186/s12935-020-01517-z.
2. Bruneau, S., Datta, D., Flaxenburg, J.A., Pal, S., and Briscoe, D.M. (2012). TRAF6 inhibits proangiogenic signals in endothelial cells and regulates the expression of vascular endothelial growth factor. Biochem Biophys Res Commun *419*, 66-71. 10.1016/j.bbrc.2012.01.128.
3. Kolesnichenko, M., Mikuda, N., Hopken, U.E., Kargel, E., Uyar, B., Tufan, A.B., Milanovic, M., Sun, W., Krahn, I., Schleich, K., et al. (2021). Transcriptional repression of NFKBIA triggers constitutive IKK- and proteasome-independent p65/RelA activation in senescence. EMBO J *40*, e104296. 10.15252/embj.2019104296.
4. Liu, M., Huang, L., Liu, Y., Yang, S., Rao, Y., Chen, X., Nie, M., and Liu, X. (2023). Identification of the MMP family as therapeutic targets and prognostic biomarkers in the microenvironment of head and neck squamous cell carcinoma. J Transl Med *21*, 208. 10.1186/s12967-023-04052-3.
5. Santoro, R., Zanotto, M., Simionato, F., Zecchetto, C., Merz, V., Cavallini, C., Piro, G., Sabbadini, F., Boschi, F., Scarpa, A., and Melisi, D. (2020). Modulating TAK1 Expression Inhibits YAP and TAZ Oncogenic Functions in Pancreatic Cancer. Mol Cancer Ther *19*, 247-257. 10.1158/1535-7163.MCT-19-0270.
6. Chen, J., Zhang, N., Wen, J., and Zhang, Z. (2017). Silencing TAK1 alters gene expression signatures in bladder cancer cells. Oncol Lett *13*, 2975-2981. 10.3892/ol.2017.5819.
7. Rong, C., Muller, M.F., Xiang, F., Jensen, A., Weichert, W., Major, G., Plinkert, P.K., Hess, J., and Affolter, A. (2020). Adaptive ERK signalling activation in response to therapy and in silico prognostic evaluation of EGFR-MAPK in HNSCC. Br J Cancer *123*, 288-297. 10.1038/s41416-020-0892-9.
8. Pickard, A., Amoroso, F., McCulloch, K., Erickson, A., Sachdeva, A., Steele, R., Mukherjee, D., Dellett, M., McComb, J., McCaffery, L., et al. (2023). IRF7 impacts on prostate cancer cell survival in response to radiation. bioRxiv, 2022.2009.2023.509205. 10.1101/2022.09.23.509205.

9. Mesquita, F.P., Moreira-Nunes, C.A., da Silva, E.L., Lima, L.B., Daniel, J.P., Zuerker, W.J., Brayner, M., de Moraes, M.E.A., and Montenegro, R.C. (2020). MAPK14 (p38alpha) inhibition effects against metastatic gastric cancer cells: A potential biomarker and pharmacological target. Toxicol In Vitro *66*, 104839. 10.1016/j.tiv.2020.104839.
