# Supplementary material for: Simultaneous CRISPR screening and spatial transcriptomics reveal intracellular, intercellular, and functional transcriptional circuits
Source: Cell. Author manuscript; Available in PMC 2025 Jun 4. (PMC12135205; doi:10.1016/j.cell.2025.02.012)
Supplement: 1 [file NIHMS2080922-supplement-1.pdf]

CellPress
OPEN ACCESS

Supplemental figures

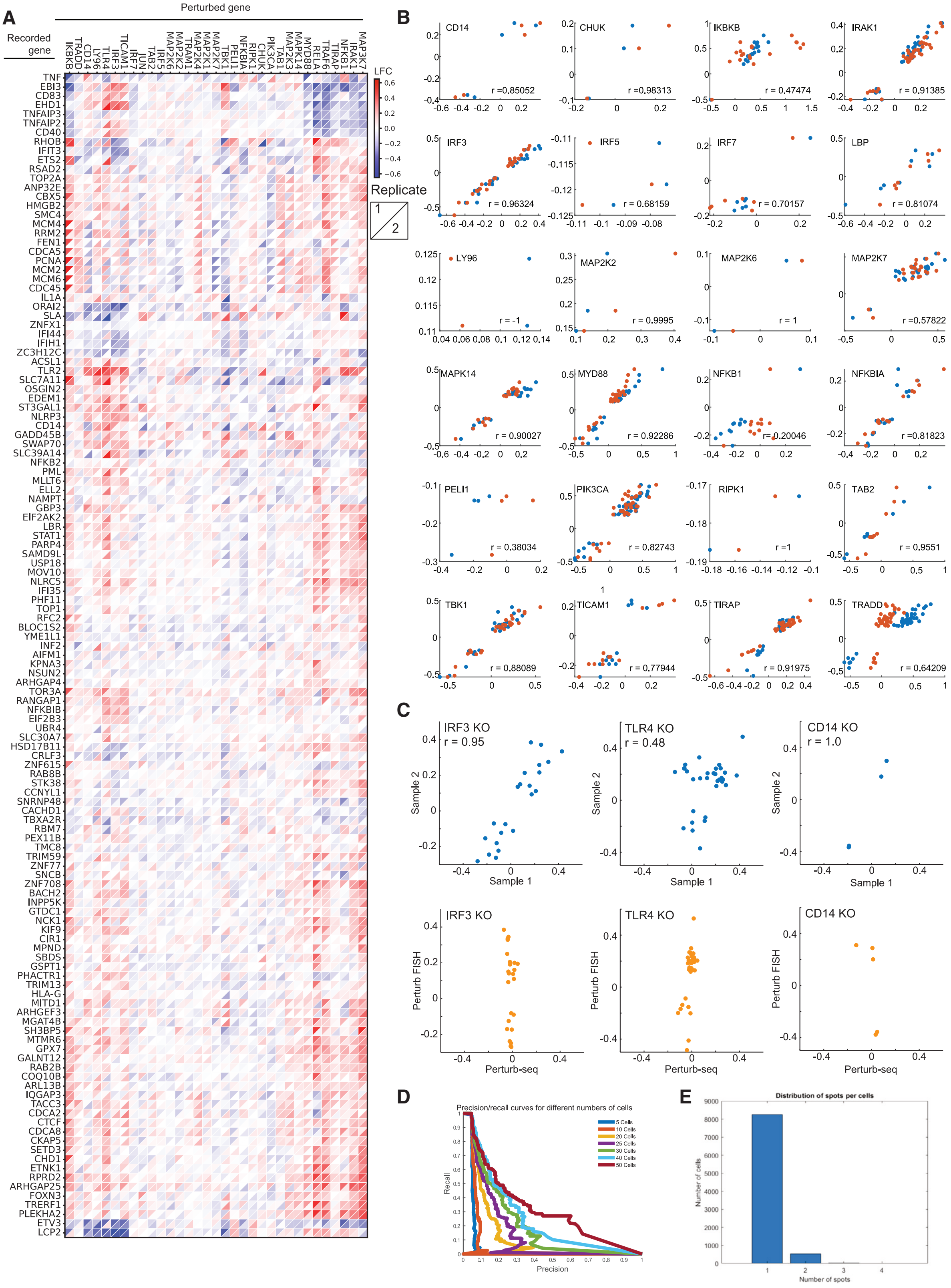

(legend on next page)

Figure S1. Consistency of Perturb-FISH results and power analysis, related to Figure 3

(A) Heatmap of LFC effect sizes (color bar) between Perturb-FISH replicate 1 (upper left triangle of each square) and replicate 2 (lower right triangle). Rows and columns are clustered based on UPGMA clustering of sample1 data.

(B) Scatterplots of effects for each targeted gene comparing the effects of the two gRNAs (y axis, red and blue) vs. all cells (x axis). Insets show correlations between gRNAs.

(C) Left: scatterplots of significant effect sizes ($q < 0.1$) determined in Perturb-seq (x axis) and combined Perturb-FISH (y axis). Right: scatterplots of significant effect sizes ($q < 0.1$) determined in Perturb-FISH replicate 2 (x axis) and replicate 1 (y axis). Shown are the effects from IRF3, TLR4, and CD14, which have the lowest correlation between Perturb-FISH and Perturb-seq. Effects represent log-fold changes (LFCs; natural log base) in expression relative to control cells.

(D) Precision recall curves for effects learned in downsampled Perturb-FISH data using increasing numbers of cells.

(E) Distribution of number of spots per cell with at least a spot.

CellPress
OPEN ACCESS

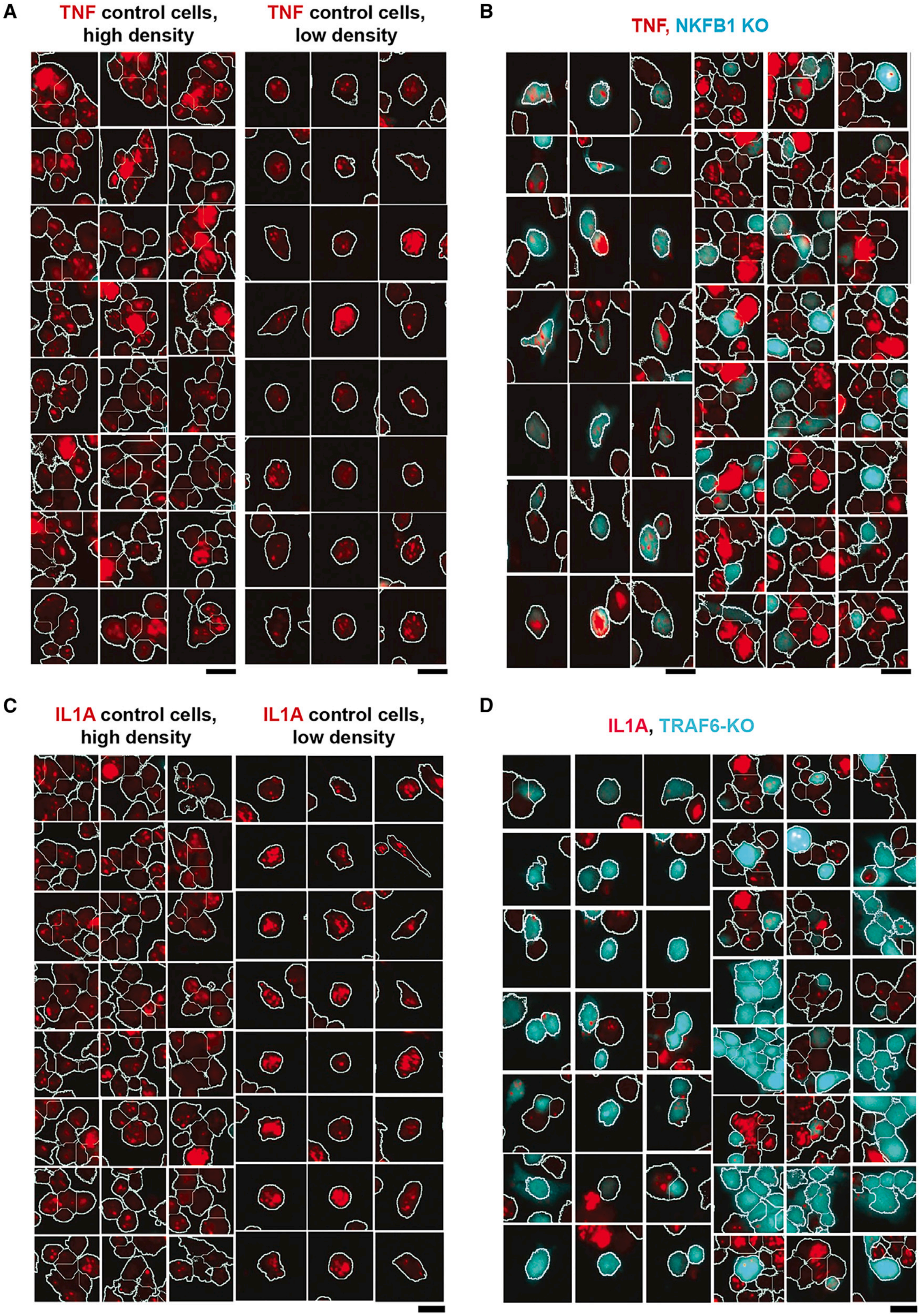

(legend on next page)

Figure S2. Low-plex validation of density-related effects from perturbations on *TNF* and *IL1A* expression, related to Figure 4

(A) RNAscope images showing TNF mRNAs (in red) in THP1s cells at low and high density.

(B) Same as (A) but now showing blue cells received a guide against *NFKB1*, other cells received a non-targeting guide. Scale bar: 30 μm.

(C and D) Same as (A) and (B) but for *IL1A* measurement and *TRAF6* knockout.

CellPress
OPEN ACCESS

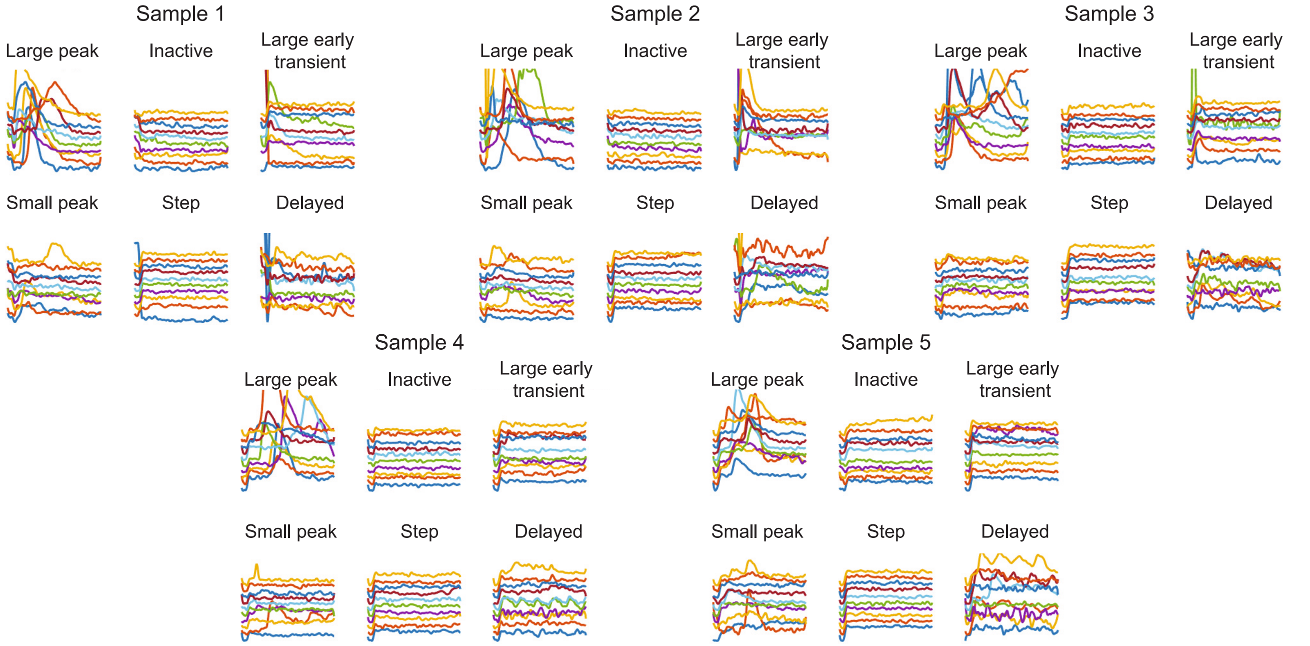

Figure S3. Example traces of calcium activity between five experimental replicates showing the consistency of detected phenotypes, related to Figure 5

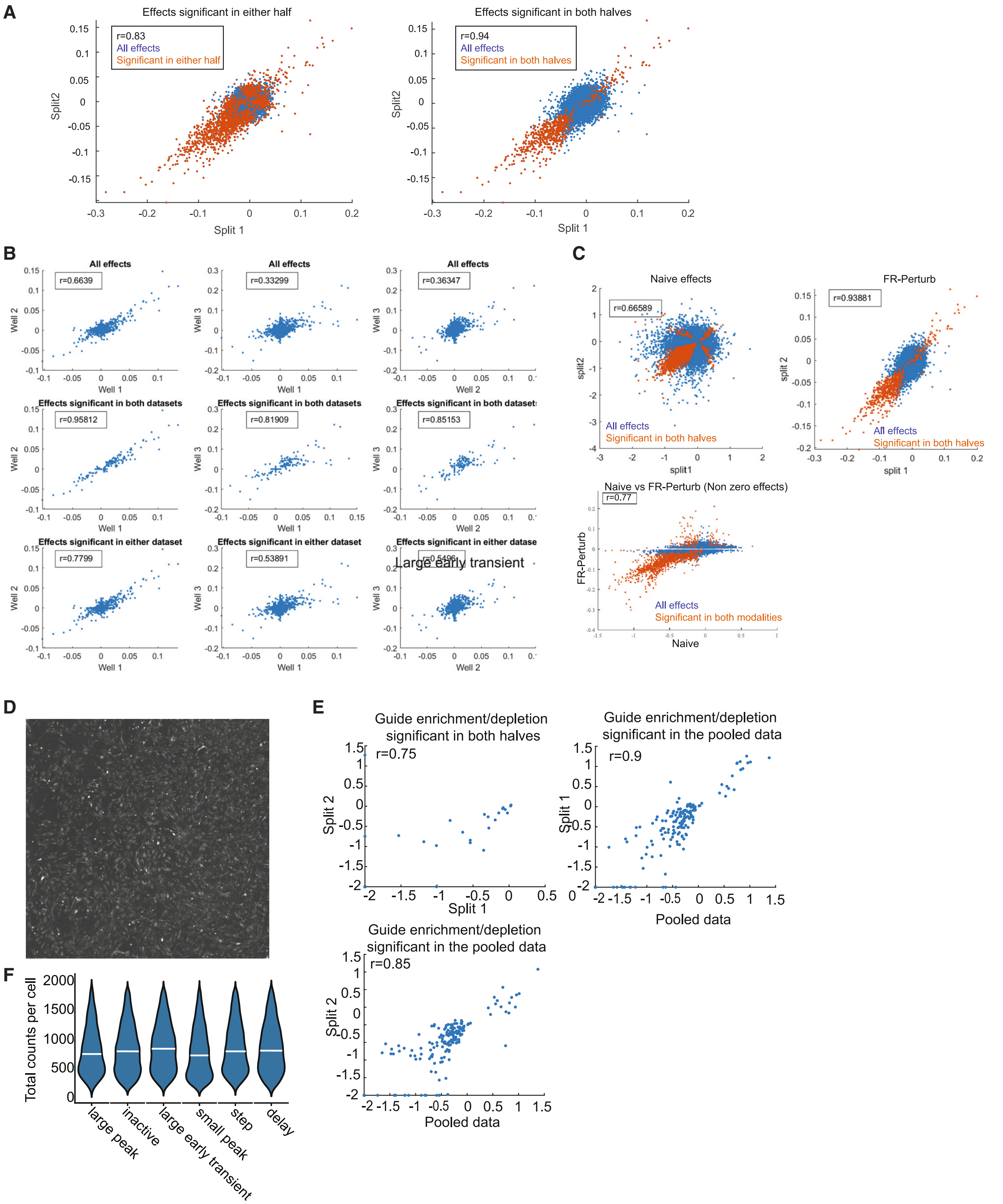

Figure S4. Consistency of the effects of gRNA perturbations on gene expression and functional phenotypes in astrocytes, related to Figure 5
(A) Scatterplots of gRNA perturbation effects in random subsets of the data. Blue: all effects. Orange: effects significant in one half left or both halves (right) of the data.

(legend continued on next page)

(B) Scatterplots highlighting the consistency of the results between three biological replicates: well 2 vs. well 1 (left column), well 3 vs. well 1 (center), well 3 vs. well 2 (right column). All effects are shown on the first row, only effects significant in both replicates are shown in the center row, and effects significant in either dataset are shown in the bottom row.
(C) Scatterplots comparing the size of effects inferred by FR-Perturb with effects inferred from a "naive approach" (STAR Methods), and their consistency when randomly sub-setting the dataset in 2 and comparing effects from each half.
(D) Representative image of Fluo4 signal (488 nm) showing the morphology of astrocytes.
(E) Scatterplots showing the consistency of the perturbation enrichment analysis between biological replicates.
(F) Violin plots showing the difference in total detected transcripts in cells of different calcium phenotypes.

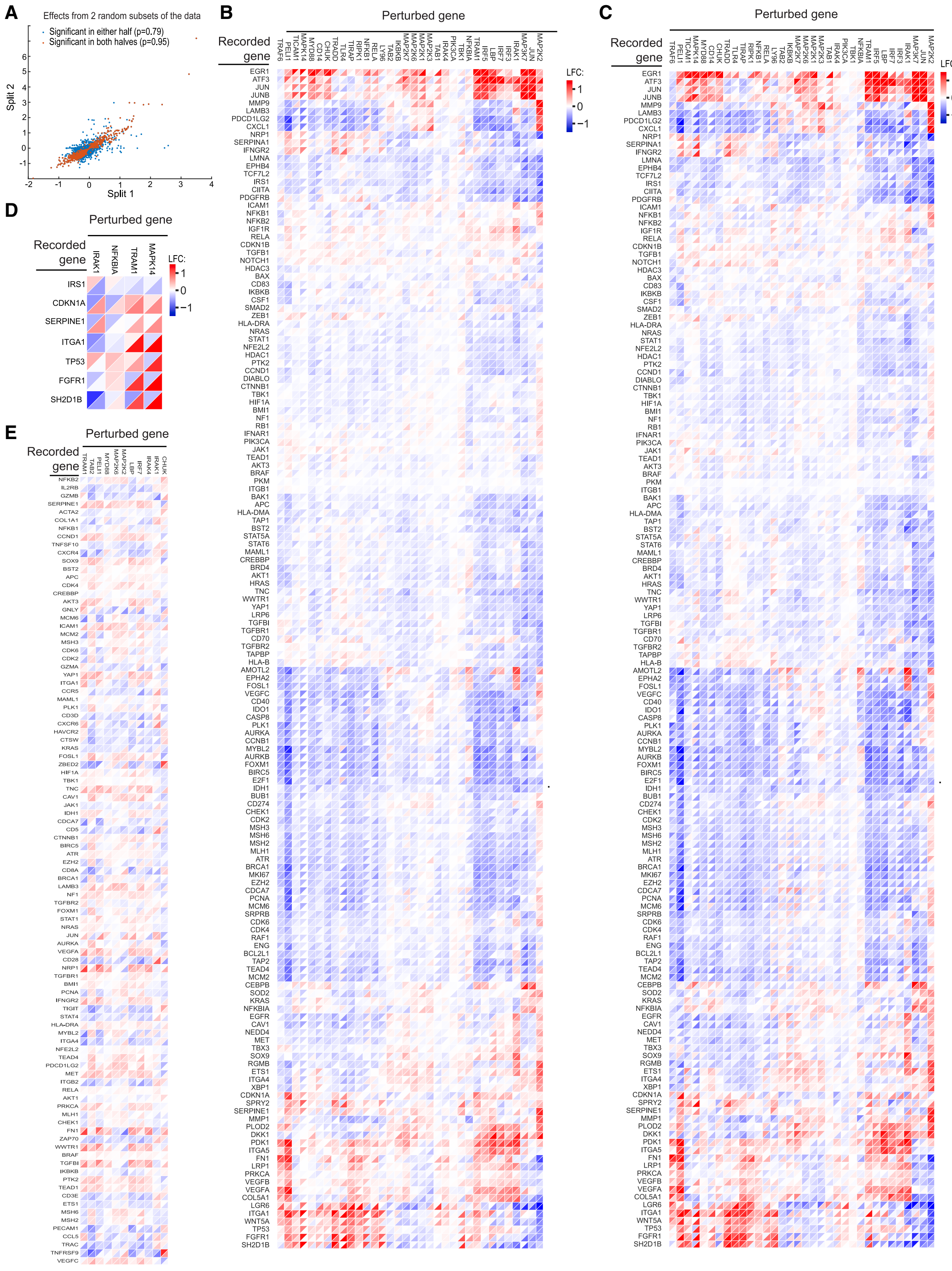

(legend on next page)

CellPress
OPEN ACCESS

Figure S5. Consistency of results from gRNA perturbations in the tumor xenograft, and comparison between neighborhood compositions, related to Figure 6

(A) Scatterplot showing the consistency of LFC effects between random subsets of the tumor xenograft perturbation data. Blue: effects significant in either half, orange: effects significant in both.

(B) Heatmap comparing LFC (color bar) effects of gRNA perturbation (x axis) on gene expression (y axis) in two random subsets (bottom left vs. top right of each square) of the data.

(C) Heatmap comparing LFC (color bar) effects of gRNA perturbations (x axis) on gene expression (y axis) in tumor cells with (top left) and without (bottom right) a T cell neighbor.

(D) Same heatmap focused on the significant effects that are different between conditions.

(E) Heatmap comparing LFC effects (colorbar) of gRNA perturbation in tumor cells (x axis) on gene expression (y axis) in two random subsets (bottom right and top left of each square) of the population of T cells.

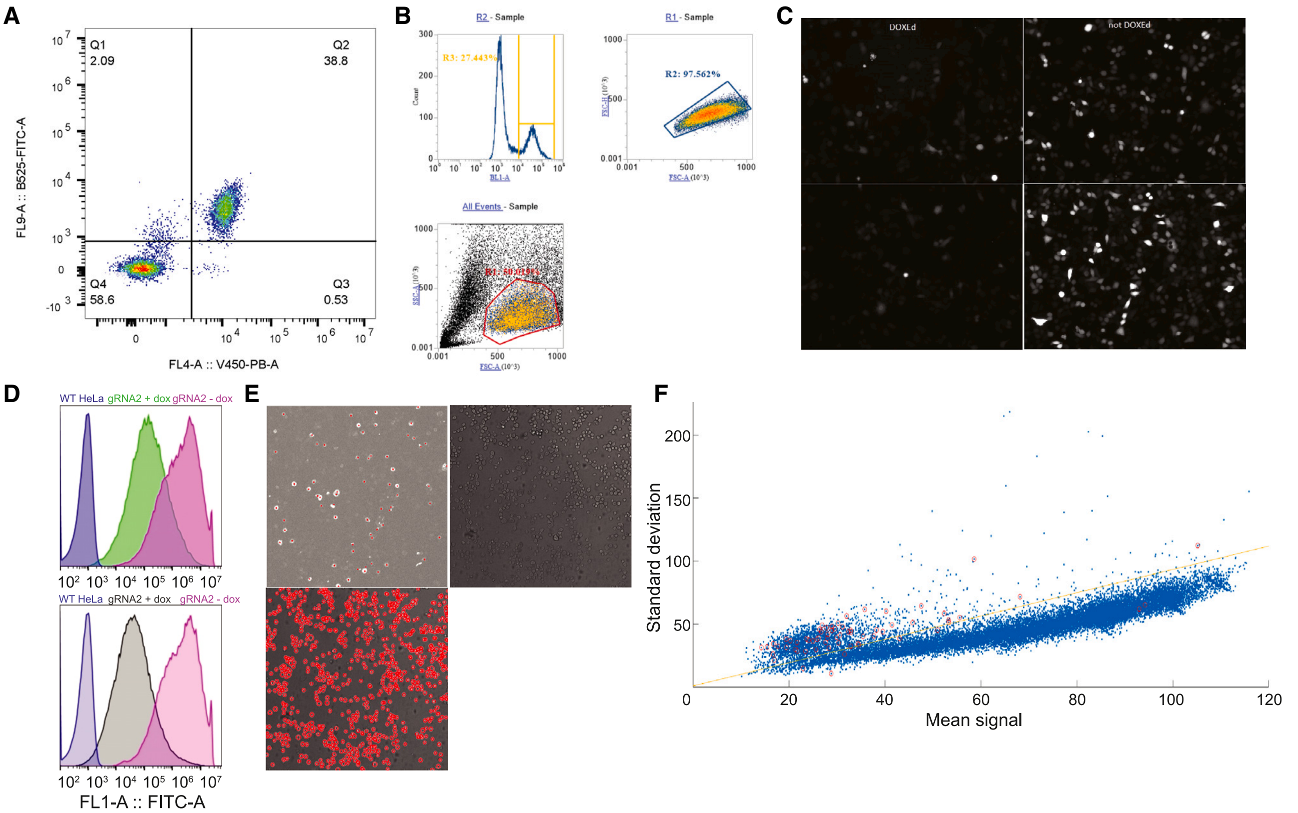

Figure S6. Quality control of Cas9 performance and decoding, related to STAR Methods

(A) Flow cytometry validation of engraftment of PBMCs in mouse peripheral blood. Cells were stained with FITC anti-human CD3 (y axis) and Brilliant Violet 421 anti-human CD45 (x axis).

(B) Evaluation of Cas9 activity. Flow cytometry report of THP1 cells infected with a vector containing both a GFP sequence and a guide against GFP under control of a canonical U6 promoter. 73% of cells are GFP negative (top left).

(C) Images of HELA cells expressing GFP, Cas9, and a U6T7 gRNA against GFP.

(D) Flow cytometry report showing the efficiency of the KO on cells from (B).

(E) Images showing the detection of positive and negative cells from images in 488 and bright field used to validate the efficiency of the knockout.

(F) Scatterplot of decoded guides showing standard deviation (y axis) as a function of mean signal across all 15 images (x axis). Dots circled in red indicate false positives (blank barcodes that do not actually correspond to any guide in the library). The line indicates the applied cutoff used during quality filtering of the data. Only decoded guides below the cutoff line were used in downstream analysis.
